# Bioinspired Structural Design Enables Synergistic Toughness and Conductivity in Hydrogels for Advanced Wearable Electronics

**DOI:** 10.1007/s40820-026-02094-y

**Published:** 2026-02-09

**Authors:** Yi Liu, Xuchen Wang, Junjie Wang, Zhuang Li, Kelong Ao, Guangwei Liang, Haiqing Liu, Qirui Zhang, Mengjiao Pan, Dahua Shou

**Affiliations:** 1 Future Intelligent Wear Centre, School of Fashion and Textiles, The Hong Kong Polytechnic University, Hung Hom, Kowloon, Hong Kong China; 2 School of Intelligent Manufacturing and Smart Transportation, Suzhou City University, Suzhou, 215104 Jiangsu China; 3 Research Centre of Textiles for Future Fashion, The Hong Kong Polytechnic University, Hung Hom, Kowloon, Hong Kong China; 4 Research Institute for Intelligent Wearable Systems, The Hong Kong Polytechnic University, Hung Hom, Kowloon, Hong Kong China; 5 PolyU-Xingguo Technology and Innovation Research Institute, The Hong Kong Polytechnic University, Hung Hom, Kowloon, Hong Kong China

**Keywords:** Conductive hydrogel, Bioinspired design, Mechanical–electrical synergy, Wearable electronics, Gesture recognition

## Abstract

Conductive hydrogels are revolutionizing the fields of wearable sensors, implantable bioelectronics, and soft robotics. However, achieving both mechanical robustness and high conductivity within a single system remains challenging. Here, inspired by the cooperative vascular–neural networks in biological tissues, we develop a nanofiber-reinforced conductive hydrogel composed of poly(vinyl alcohol) (PVA), aramid nanofibers (ANFs), and in situ polymerized PEDOT:PSS. Through solvent- and thermally induced structural reorganization, the hydrogel evolves into a bi-continuous architecture in which the mechanical and conductive networks are intimately coupled. The tough, ANF-reinforced porous PVA mimics the vascular system, providing mechanical support and maintaining toughness, while the poly(3,4-ethylenedioxythiophene) (PEDOT) network resembles neural pathways, enabling efficient electron transport. This structural evolution enables a rare synergy of high tensile strength (10.72 MPa) and ultrahigh conductivity (452.75 S m^−1^) with excellent biocompatibility. The hydrogel maintains stable conduction under impact and complex deformation, supporting multimodal sensing from large-amplitude joint motion to low-amplitude electrophysiological signals: electrocardiographic and electromyographic. When integrated with a convolutional neural network, it achieves 99.54% accuracy in recognizing five complex hand gestures. This bioinspired strategy paves the way for developing robust and conductive hydrogels toward next-generation intelligent wearable electronics.

**Supplementary Information:**

The online version contains supplementary material available at 10.1007/s40820-026-02094-y.

## Highlights

- A bioinspired design mimicking the cooperative vascular–neural networks in biological tissues was proposed to guide the development of conductive hydrogels.
- Solvent- and thermally induced structural reorganization enhances poly(vinyl alcohol) crystallinity and poly(3,4-ethylenedioxythiophene) chain alignment, yielding a synergistic combination of high tensile strength (10.72 MPa) and ultrahigh conductivity (452.75 S m^−1^).
- The hydrogel ensures stable electrical conduction and reliable multimodal sensing, enabling accurate and electromyographic/electrocardiographic monitoring and 99.54% gesture recognition accuracy.

**Supplementary Information:**

The online version contains supplementary material available at 10.1007/s40820-026-02094-y.

## Graphical abstract

**Figure:**
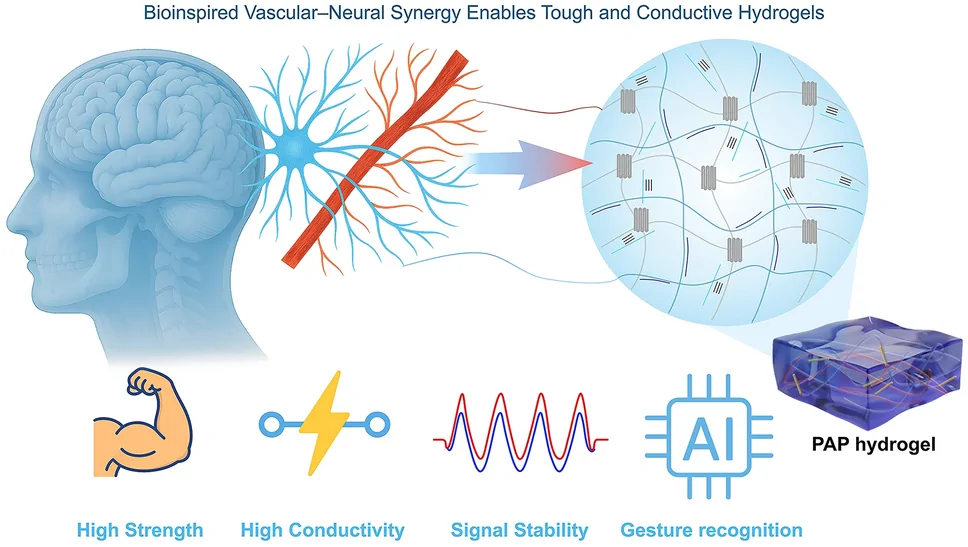

## Introduction

Over millions of years of evolution, nature has perfected the mechanisms for achieving robustness and functionality through hierarchical structural organization. From the layered architecture of bones and nacre to the interconnected neural and vascular networks in biological tissues, these archetypal systems exemplify how hierarchical, multiscale structure governs complex functions. Despite their compositional diversity, all biological systems share an underlying essence: Hierarchical structural organization can achieve synergistic performance that overcomes conventional trade-offs [1].

Although nature has perfected structural evolution over millions of years, realizing such hierarchical coordination in synthetic soft systems remains largely unexplored. Among soft materials, conductive hydrogels have attracted increasing attention due to stretchability, biocompatibility, and the capacity to sustain signal transmission under mechanical deformation [2–5]. However, the development of these materials faces a fundamental design conflict: Improving conductivity typically undermines mechanical integrity, and vice versa [6–8]. This incompatibility arises from the disparate structural demands of electronic and mechanical functions. For instance, ionically conductive hydrogels typically exhibit limited conductivity [9], while electronically conductive systems offer higher electrical performance but often remain mechanically fragile [10, 11]. Enhancing conductivity via rigid conductive fillers frequently compromises the structural integrity and elasticity of the matrix [12, 13], as seen in soft conductive systems incorporating conducting polymer networks [14], silver nanowires (AgNWs) electronic meshes [15], or MXene-based hybrid conductive frameworks [16, 17], whose highly percolated networks provide conductivities of 10^2^–10^4^ S m^−1^ but severely restrict deformability. Conversely, reinforcing the network through polymer chain entanglement, secondary crosslinking or nanofiber-reinforced hybrid architectures [18, 19], can interrupt conductive networks or diminish charge carrier mobility [20–22]. Even advanced microstructural engineering, including aligned or anisotropic architectures, may enhance directional stiffness or charge transport yet still fails to overcome the intrinsic mechanical–electrical coupling conflict [23, 24]. As a result, integrating both high conductivity and mechanical robustness within a single hydrogel system remains a significant challenge, with most reported materials favoring one functionality at the expense of the other.

Among various conductive fillers such as carbon nanomaterials [25], liquid metals [26], or conducting polymers [27], poly(3,4-ethylenedioxythiophene):polystyrene sulfonate (PEDOT:PSS) stands out for its high conductivity, biocompatibility, and environmental stability [4, 5, 28–30]. Nevertheless, its core–shell morphology, where conductive PEDOT-rich domains are encapsulated by insulating PSS chains, severely limits charge transport [31]. Post-treatment strategies, such as solvent doping (*e.g.,* DMSO), acid treatment, and thermal annealing, can significantly enhance conductivity by promoting chain rearrangement and phase separation, yet often result in mechanical fragility [11, 32–34]. Blending PEDOT:PSS with flexible polymers is commonly used to improve stretchability, but the simple physical mixing typically disrupts the conductive network and dilutes the conductive phase [35–38]. This inherent trade-off underscores the persistent challenge of achieving both mechanical toughness and high electrical performance in PEDOT:PSS-based hydrogels.

To overcome this intrinsic conflict, we turned to biological systems for inspiration. Inspired by the brain’s vascular–neural system, in which blood vessels not only provide mechanical support and nutrient delivery but also work synergistically with neural circuits to facilitate signal transmission, we developed a bioinspired structural design strategy that translates the cooperative principles of biological networks into hydrogels. Through solvent- and thermally induced structural reorganization, a PVA/ANF(aramid nanofiber)/PEDOT:PSS hydrogel featuring an interpenetrating bi-continuous phase was constructed. Within this architecture, ANFs provide structural confinement that guides the formation of a PEDOT-rich network. The subsequent DMSO/H₂O exchange and thermal annealing promote PEDOT chain rearrangement, partial removal of insulating PSS, and enhanced PVA crystallinity. This bioinspired design overcomes the long-standing trade-off, enabling simultaneous enhancement of mechanical strength (tensile strength of 10.72 MPa) and ultrahigh conductivity (452.75 S m^−1^), outperforming the most previously reported PVA/PEDOT:PSS-based conductive hydrogels. Benefiting from its stable conduction and excellent biocompatibility, the PAP hydrogel is well suited for stable monitoring of human motion and low-amplitude electrocardiographic (ECG), and electromyographic (EMG) signals. When integrated with a convolutional neural network (CNN)-based EMG interface, the hydrogel enabled a multi-channel gesture recognition system with 99.54% accuracy across five gestures, demonstrating its promise for intelligent wearable electronics. This bioinspired framework bridges biological systems and soft electronics, paving the way for materials that combine robustness, conductivity, and intelligence.

## Experimental Section

### Materials

Poly(vinyl alcohol) (brand type 1799, 74.9 kg mol^−1^, alcoholysis degree of 98% ~ 99%), 3,4-ethylenedioxythiophene (EDOT), dimethyl sulfoxide (DMSO), potassium hydroxide (KOH), and n-Hexane were purchased from Macklin Biochemical Technology Co., Ltd. (Shanghai, China). Kevlar® aramid fiber was supplied by DuPont (USA). Ammonium persulfate (APS) was purchased from Sigma-Aldrich (USA). Poly(sodium-p-styrenesulfonate) (PSSNa, average M_w_ ≈ 70,000) was purchased from Thermo Scientific (USA). All chemicals were of analytical reagent grade and used as received without further treatment.

### Preparation of Aramid Nanofiber (ANF) and PVA Solutions

Typically, 2 g Kevlar® aramid fiber was added to a mixture of 96 g DMSO, 2 g distilled water, and 2 g KOH. The mixture was stirred at room temperature for at least 7 days until a dark red 2 wt% ANF solution was obtained. Separately, PVA powder of 10 g was dissolved in 90 g distilled water at 90 °C under vigorous stirring for 4 h to prepare a 10 wt% PVA solution.

### Preparation of PVA-ANF (PA) Hydrogels

Taking P_10_A_1_ (a hydrogel formulation with a mass ratio of 10:1 between PVA and ANF) as an example, 8 g 10 wt% PVA solution mixed with 4 g 2 wt% ANF solution under 90 °C. After degassing, the homogeneous mixture was poured into a PTFE mold and placed on a foam platform inside a sealed thermostatic water bath at 37 °C, allowing the solution to slowly absorb water vapor from the surrounding air. After 30 min, the hydrogel precursor was subjected to three freezing–thawing cycles followed by solvent exchange in deionized (DI) water to obtain P_10_A_1_ hydrogels.

### Preparation of Non-Annealed PVA/ANF/PEDOT:PSS (PAP-n) Hydrogels

A 0.1 M aqueous solution of ammonium persulfate (APS) and poly(sodium *p*-styrenesulfonate) (PSSNa) was sonicated for 10 min to ensure complete dissolution and obtain a homogeneous solution. The PA hydrogels were soaked in this solution for 24 h to allow sufficient diffusion. Next, various concentrations of 3,4-ethylenedioxythiophene (EDOT) monomer were added to n-hexane and stirred thoroughly for uniform EDOT solutions. The pretreated PA/APS-PSSNa samples were then soaked in EDOT/n-hexane solution for 48 h at room temperature. In this process, EDOT monomers diffused from the n-hexane phase into the APS-PSSNa preloaded hydrogel and were oxidatively polymerized in situ to form the conductive PEDOT:PSS network within the gel. After polymerization, the gels were washed with ethanol and DI water several times to remove unreacted components and subsequently swollen in DI water for 2 days. The results were denoted as PAP-n gels, where *n* indicates unannealed samples.

### Preparation of Annealed PVA/ANF/PEDOT:PSS (PAP) Hydrogels

PAP-n gels were immersed in the co-solvent mixture of DMSO (13 wt%) and H_2_O (87 wt%) at 37 °C for 24 h, followed by uniform drying at 30 °C for 24 h between two glass substrates. Subsequently, the samples underwent three cycles of annealing at 130 °C for 30 min each and were then swollen in deionized water for 2 days. The final products were denoted as P_x_A_y_P_z_ hydrogels, where P_x_A_y_P_z_ represents a hydrogel formulation, x:y represents the mass ratio of PVA to ANF, and z denotes the EDOT precursor content.

## Results and Discussion

### Processing and Multifunctionality of the PAP Hydrogel

**Fig. 1:**
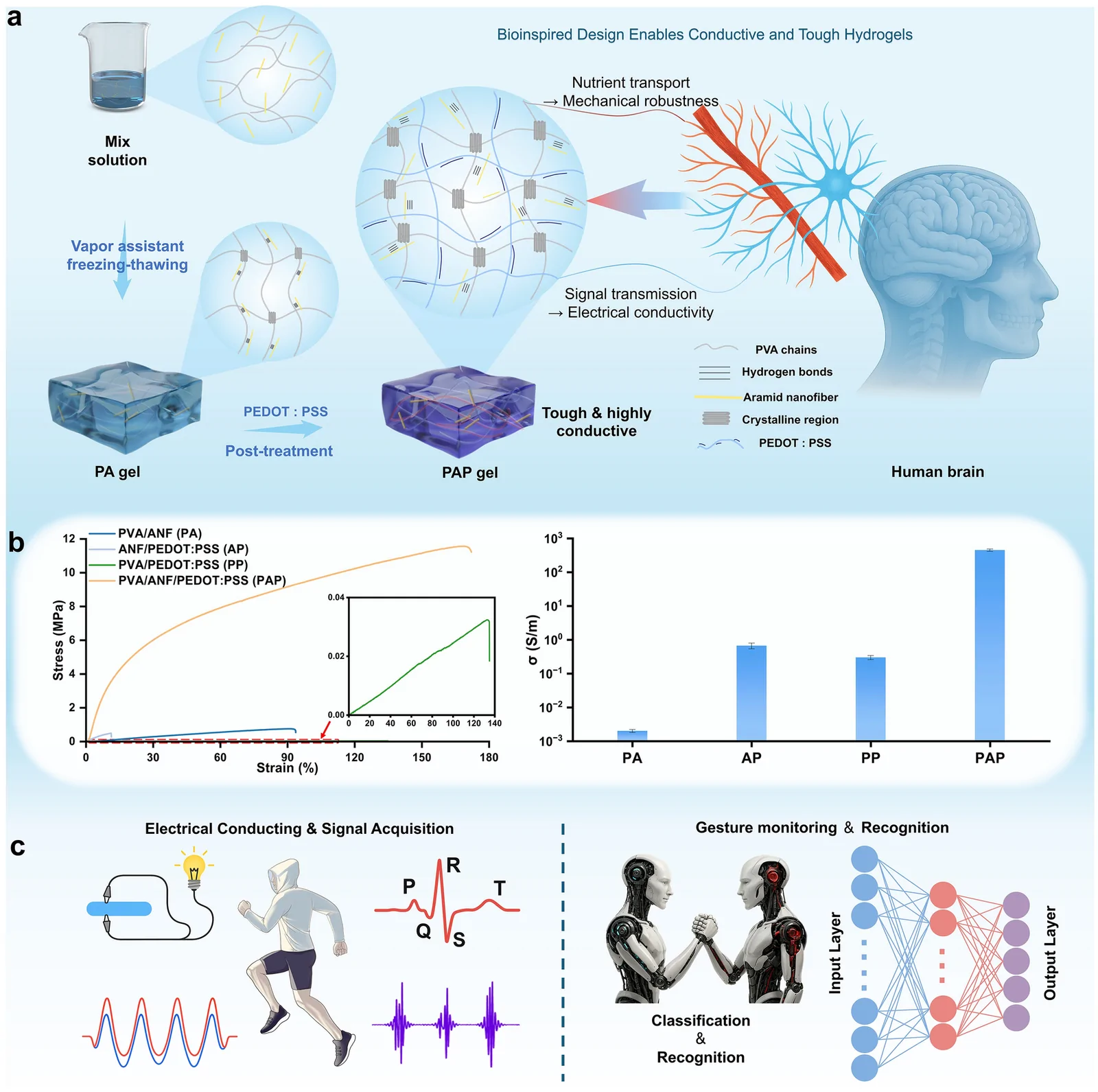
Design and fabrication, performance enhancement, and multifunctional applications of the PAP hydrogel. **a** Bioinspired design and fabrication process of the PAP hydrogel. **b** Comparison of mechanical and electrical properties among PA, AP, PP, and PAP hydrogels. **c** Application schematic of the PAP hydrogel sensor

Following this rationale, a stepwise fabrication route was established for PAP hydrogel (Fig. 1a). Initially, a homogeneous solution was prepared by mixing 10 wt% PVA and 2 wt% ANFs in DMSO. To prevent ANF aggregation in aqueous environments, a vapor-assisted approach was employed, in which slow water vapor diffusion into the DMSO-based solution induced pre-gelation, preserving nanofiber dispersion and stabilizing the mixture for subsequent processing. The freeze–thaw process then promoted partial PVA crystallization and network formation, during which ANFs were uniformly embedded and anchored via interfacial hydrogen bonding, resulting in a porous hydrogel network. Scanning electron microscopy (SEM) imaging of the freeze-dried PVA-ANF gel (Fig. S1a) confirmed this open and interconnected morphology, which provides abundant space and anchoring sites for subsequent PEDOT:PSS infiltration. Subsequently, the gel was sequentially soaked in an aqueous solution containing polystyrene sulfonate (PSS) and oxidant, followed by an EDOT-containing organic phase to initiate in situ oxidation polymerization. This process enables uniform growth of PEDOT chains within the porous matrix, forming a structurally integrated conductive network. A final DMSO/H_2_O exchange and thermal annealing further optimized the hierarchical structure by promoting PEDOT chain alignment, partially reducing the insulating PSS content, and enhancing PVA crystallinity, exhibiting a markedly denser network (Fig. S1b), thus yielding a bi-continuous and well-integrated conductive framework. This hierarchical construction mimics the cooperative development of vascular and neural networks in biological tissues, where the ANF-reinforced PVA framework provides vascular-like mechanical support, and the subsequently formed PEDOT:PSS network serves as neural-like conductive pathways for efficient signal transmission. As a direct outcome of this hierarchical structural strategy, the PAP hydrogel exhibited remarkable breakthroughs in both mechanical and electrical performance. As shown in Fig. 1b, we compared the mechanical and electrical properties of PVA/ANF (PA), ANF/PEDOT:PSS (AP), PVA/PEDOT:PSS (PP), and the integrated PVA/ANF/PEDOT:PSS (PAP) hydrogel, while the full mechanical metrics (*E*, *σ*_*b*_, and *W*) and the corresponding water content comparison are provided in Fig. S2. PAP delivers substantially enhanced mechanical and electrical performance compared with all control samples, demonstrating a post-treatment enabled synergy between the ANF/PVA reinforcing framework and the PEDOT:PSS conductive pathways. Specifically, compared to the PP hydrogel without ANFs and post-treatment, the tensile strength increased from 0.034 to 10.72 MPa, toughness from 0.03 to 12.80 MJ m^−3^, and Young’s modulus from 0.02 to 29.89 MPa. More strikingly, the conductivity rose dramatically from 0.298 to 452.75 S m^−1^ (over 1500-fold), highlighting the effectiveness of the synergistic design in addressing the long-standing trade-off between mechanical robustness and high conductivity in conductive hydrogel systems. Benefiting from these superior properties, the PAP hydrogel supports stable electrical conduction and reliable monitoring of human motion and biophysical signals such as ECG and EMG. Its high-fidelity signal acquisition also enables machine learning-assisted gesture recognition using CNNs, demonstrating its promise for next-generation wearable electronics and intelligent interfaces (Fig. 1c).

### Mechanical Performance of the PAP Hydrogel

**Fig. 2:**
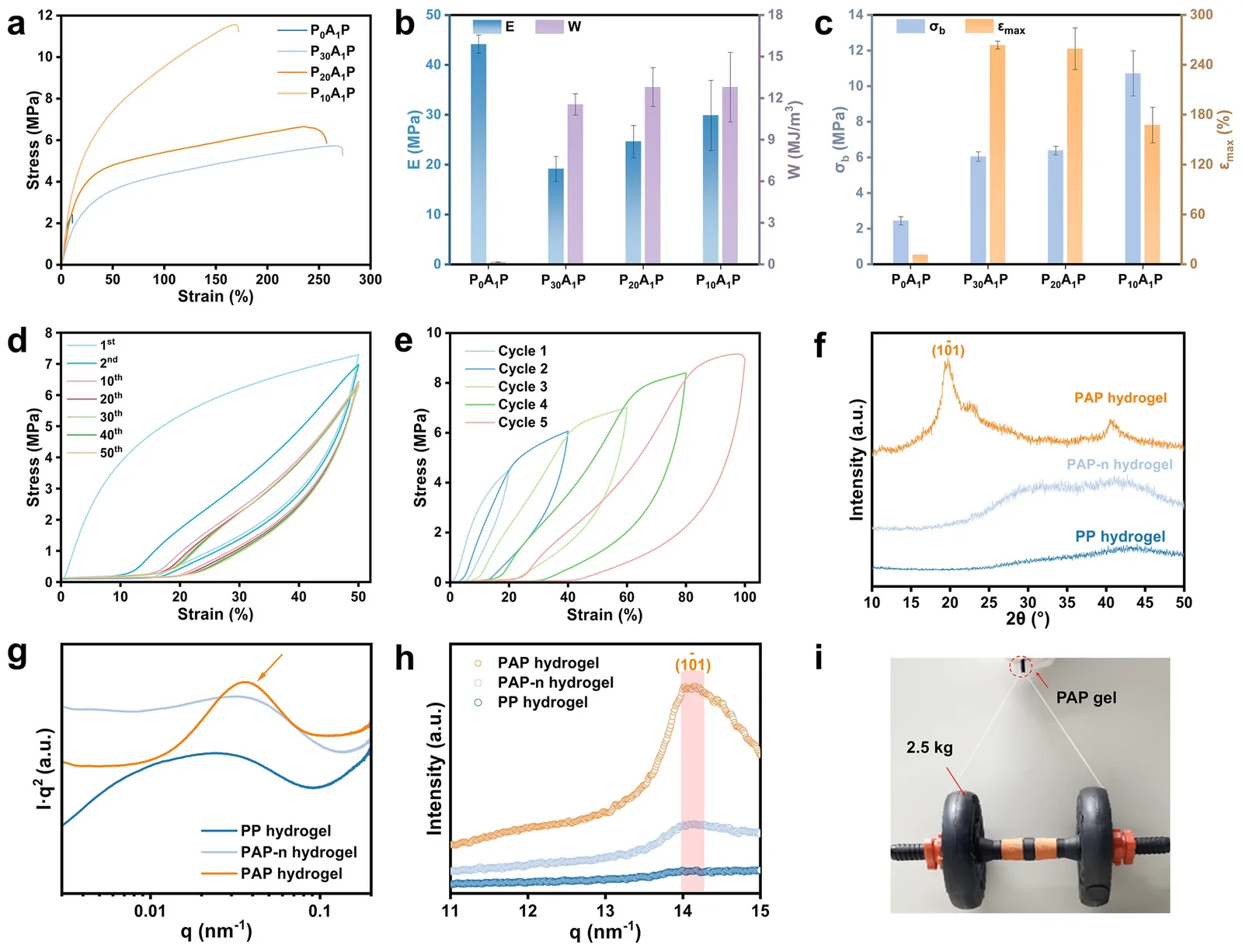
Mechanical performance and enhancement mechanism in PAP hydrogel. **a** Uniaxial tensile stress–strain curves of PAP hydrogels with varying PVA/ANF ratios. **b, c** Young’s modulus (*E*), toughness (*W*), fracture stress (*σ*_*b*_), and maximum strain (*ε*_*max*_) of the corresponding samples. **d, e** Cyclic tensile tests of the PAP hydrogel. **f–h** XRD, 1D SAXS and 1D WAXS patterns of PP, PAP-n (unannealed), and PAP (annealed) hydrogels, showing structural changes due to annealing. **i** Demonstration of the mechanical strength of PAP hydrogel via a weight-lifting test, highlighting its exceptional load-bearing capacity

To optimize the mechanical performance of the PAP hydrogel, we fixed the EDOT content at 10 wt% during polymerization and systematically varied the PVA-ANF mass ratio. All samples underwent the same vapor-assisted freeze–thaw cycling, in situ PEDOT:PSS polymerization, DMSO/H_2_O exchange, and thermal annealing to ensure the reliable formation of a mechanically reinforced and electrically conductive hydrogel framework. As shown in Fig. 2a-c, increasing the ANF content significantly enhanced the mechanical performance: Young’s modulus climbed from 19.14 to 29.89 MPa, toughness from 11.55 to 12.80 MJ m^−3^, and tensile strength from 6.05 to 10.72 MPa, with the P_10_A_1_P_10_ hydrogel exhibiting the most balanced performance. This improvement is attributed to the increasing density of rigid nanofibers and intensified hydrogen bonding with the PVA matrix, which together promotes efficient stress transfer throughout the network. Fourier transform infrared spectroscopy (FTIR) and mechanical data in Fig. S3 support this mechanism, showing enhanced O–H/N–H stretching and elevated modulus upon ANF incorporation. In contrast, the ANF-PEDOT:PSS hydrogel (without PVA) displayed a much higher modulus due to the intrinsic stiffness of the nanofibers, but it suffers from limited stretchability and poor network integrity. In addition, mechanical comparisons of PAP gels synthesized with different EDOT concentrations (5–15 wt%) during in situ polymerization (Fig. S4) revealed that tensile properties are insensitive to EDOT content, with the 10 wt% sample showing superior strength–elongation balance. Unless otherwise specified, all subsequent tests were performed on the optimized P_10_A_1_P_10_ hydrogel, which included DMSO/H_2_O exchange and thermal annealing.

We next evaluated the durability of this optimized system through repeated loading–unloading tests under both fixed and progressively increasing strain conditions. As shown in Figs. 2d and S5a, the sample retained over 88% of its initial strength after 50 cycles at 50% strain, with negligible permanent deformation (Fig. S6), demonstrating excellent fatigue resistance. The dissipated energy dropped dramatically in the initial few cycles and then stabilized, while stress remained nearly constant, indicating that only a limited number of crystalline domains were disrupted during tests, hence showing excellent fatigue resistance. Sequential cyclic tests at increasing strain levels (Figs. 2e and S5b) revealed expanded hysteresis loops, and the dissipation ratio gradually increased and stabilized at approximately 65%. This strain-dependent energy dissipation is due to the irreversible fracture of PVA crystalline domains within the ANF-reinforced network, allowing the hydrogel to effectively dissipate mechanical energy while preserving structural integrity.

To establish a mechanistic link between macroscale mechanical performance and molecular level structural organization, multiscale structural characterizations were conducted. The absorption band corresponding to the stretching vibration of hydroxyl groups in the PAP gel becomes broader and more intense than that in the unannealed samples (Fig. S7), indicating strengthened hydrogen bonding among PVA chains upon thermal annealing. As shown in Fig. 2f, only the annealed hydrogel exhibits sharp diffraction peaks at 2*θ* ≈ 19.5° and 41.1°, which are typical of the PVA crystal structures [39]. In contrast, the PAP-n and PP gels show much weaker and broader peaks, reflecting a significantly lower degree of crystallinity. These results suggest that thermal annealing promotes PVA chain rearrangement, facilitating the formation of more distinct and well-defined crystalline domains.

Small-angle X-ray scattering (SAXS) and wide-angle X-ray scattering (WAXS) were employed further to investigate the microstructural differences within the hydrogel network. As shown in the 1D SAXS profiles (Fig. 2g), the PAP hydrogel exhibited a distinct scattering shoulder in the low-q region at q ≈ 0.03614 nm^−1^, corresponding to an average interdomain spacing (*L*) of approximately 175 nm. This indicates a more expanded and well-organized nanostructure, resulting from network rearrangement and densification during thermal annealing. In contrast, the PP and PAP-n samples displayed no obvious scattering peaks, suggesting the absence of well-defined nanoscale organization. The 2D SAXS patterns visually confirm this difference (Fig. S8), where the PAP hydrogel presented a significantly stronger and more uniform isotropic scattering ring, while the other samples exhibited weak and diffuse signals. In the 1D WAXS profile, scattering peak at q ≈ 14.2 nm^−1^, corresponding to the (101) plane of semicrystalline PVA (Figs. 2h and S8) [40], becomes significantly more intense upon annealing. The 2D WAXS pattern of the PAP gel (Fig. S9a) shows a clear semicrystalline ring, absent in the unannealed gels. The peak deconvolution of the 1D profile (Fig. S9b) further demonstrates increasing PVA crystallinity, with an average size (*D*) of 16.49 nm calculated by Scherrer’s equation. Collectively, these results confirm that thermal annealing promotes the formation of well-defined crystalline domains, thereby enhancing nanoscale structural regularity and underpinning the observed improvements in mechanical properties. As direct visual validation, the PAP hydrogel could support a 2.5 kg weight without rupture (Fig. 2i), offering an intuitive demonstration of its enhanced mechanical strength.

### Electrical Behaviors of the PAP Hydrogel

**Fig. 3:**
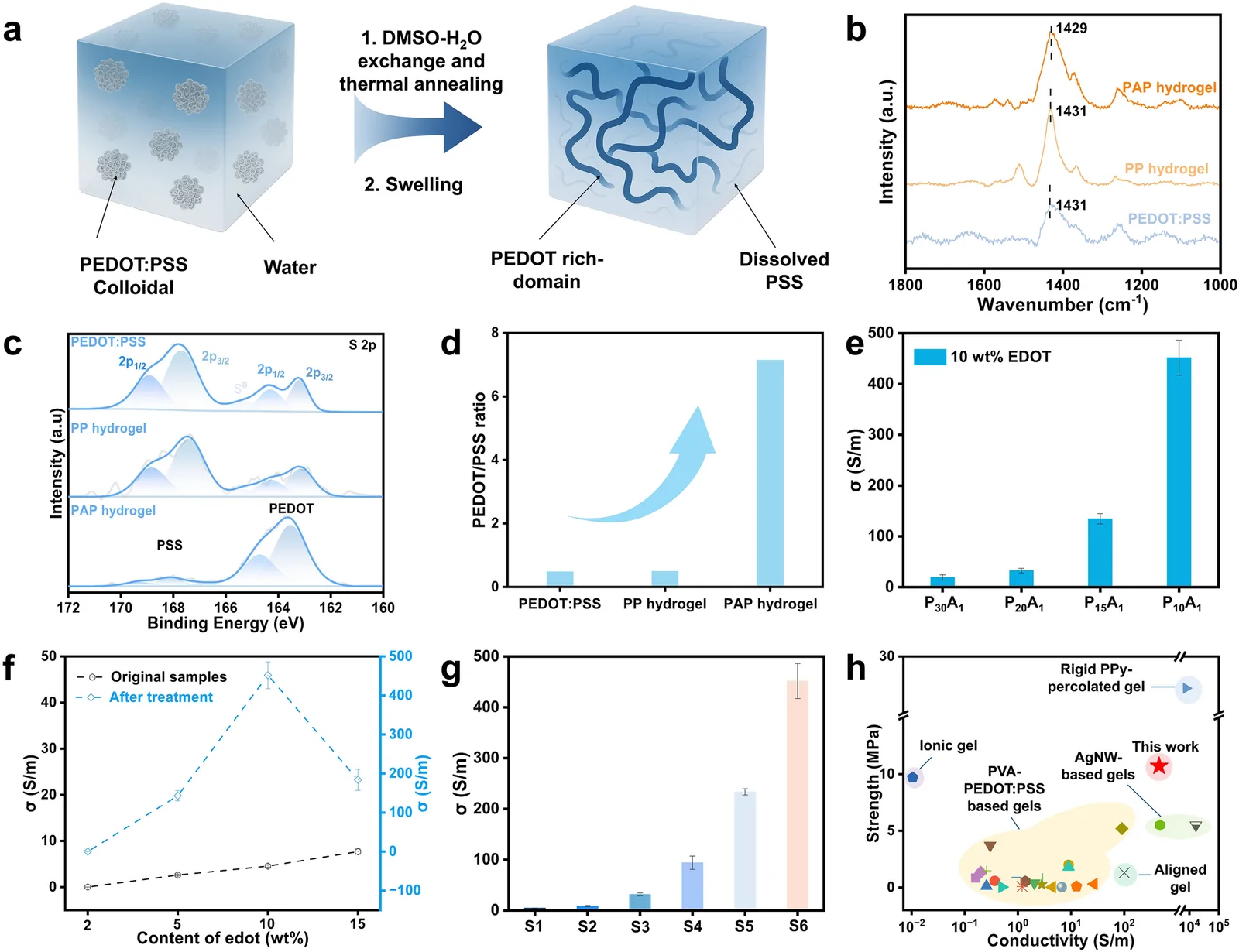
Mechanism of conductivity enhancement in PAP hydrogel. **a** Schematic illustration of PEDOT:PSS reorganization during post-treatment. **b** Raman spectra and **c** XPS spectra of different samples. **d** PEDOT/PSS ratio extracted from XPS analysis. **e** Conductivity of PAP hydrogels with varying ANF content. **f** Conductivity before and after post-treatment at different EDOT concentrations. **g** Conductivity of systematically designed hydrogels with individual processing steps omitted. S1: without annealing; S2: DMSO/H_2_O treatment only (no annealing); S3: without vapor assist; S4: without ANF; S5: annealing (without DMSO/H_2_O treatment); S6: full process. **h** Comparison of conductivity and tensile strength with reported conductive hydrogels [7, 14–16, 18, 19, 23, 24, 36, 41–58]

While thermal annealing has been shown to significantly enhance the mechanical properties of PAP hydrogels by promoting crystallinity, it also plays a vital role in reorganizing the embedded PEDOT:PSS phase. As shown in Fig. 3a, PEDOT:PSS is initially dispersed in water as colloidal aggregates, in which excess insulating PSS tightly encapsulates the conductive PEDOT chains. Upon DMSO/H_2_O solvent exchange combined with thermal annealing, the Coulombic interactions between PEDOT and PSS are disrupted, facilitating phase separation, the removal of excess PSS, and enhanced π–π stacking among PEDOT chains. Moreover, EDS sulfur mapping confirms a homogeneous distribution of the PEDOT:PSS component throughout the hydrogel (Fig. S10). This synergistic reorganization leads to the formation of continuous PEDOT-rich domains, thereby markedly improving the electron transport capability of the system.

To elucidate the mechanism underlying the conductivity enhancement of PAP hydrogel, the Raman spectrum was first performed to investigate the conformational changes of the PEDOT-rich domains. As shown in Fig. 3b, the characteristic peak at 1431 cm^−1^, corresponding to the *C*_*α*_ = *C*_*β*_ symmetric stretching vibration in PEDOT:PSS and PP hydrogel, redshifts to 1429 cm^−1^ and becomes narrower in PAP hydrogel after post-treatment. This change indicates a transformation from a benzenoid (disordered coil conformation) to a quinoid (ordered linear conformation) state, as further supported by Fig. S11. Such conformational rearrangement not only reduces carrier migration energy but also enhances π–π stacking, thereby facilitating more efficient intra- and inter-chain electron transport [7].

Further analysis was conducted to investigate the compositional evolution associated with this structural transformation. X-ray photoelectron spectroscopy (XPS) results (Fig. 3c) revealed that the S 2*p* peaks were clearly resolved into the PEDOT (161 to 166 eV) and PSS (166 to 170 eV) [33]. Compared to the PEDOT:PSS and untreated PP hydrogel, the PAP hydrogel exhibits a markedly increased PEDOT signal and a reduced PSS signal, indicating that post-treatment promotes the removal or redistribution of excess PSS and leads to a PEDOT-rich domain. This trend is further supported by atomic force microscopy (AFM), which revealed a notable increase in surface roughness and heterogeneity in PAP hydrogel (Fig. S12), consistent with phase separation between PEDOT and PSS. Additionally, water contact angle measurements (Fig. S13) showed a significant increase in surface hydrophobicity after post-treatment. As shown in Fig. 3d, the PEDOT/PSS peak area ratio increased substantially from 0.48 in PEDOT:PSS and 0.49 in the PP hydrogel to 7.14 in the PAP hydrogel. These structural and compositional changes collectively support the formation of a continuous, highly conductive PEDOT-rich pathway.

We systematically explored how variations in formulation and post-treatment affect conductivity by a standard two-probe method with a digital multimeter and four-probe method (Figs. S14 and S15), aiming to clarify the impact of the PEDOT-rich interconnected structure, as evidenced by predominantly electronic charge transport in the PAP hydrogels (Fig. S16) [59]. As shown in Fig. 3e, as the PVA-ANF mass ratio was adjusted from 30:1 to 10:1, hydrogel conductivity improved considerably from 19.0 to 452.75 S m^−1^. This sharp escalation reflects the structural regulation introduced by ANF: The more interconnected PVA-ANF network facilitates the diffusion and polymerization of PEDOT:PSS throughout the matrix. Simultaneously, ANF incorporation helped suppress excessive PVA crystallization during annealing, preserving continuous conductive pathways and enabling more efficient charge transport. In addition to ANF content, the concentration of EDOT during polymerization also plays a crucial role in determining final conductivity, as illustrated in Fig. 3f. Without post-treatment, conductivity rose only slightly from 0.01 (2 wt% EDOT) to 7.70 S m^−1^ (15 wt%) due to the intrinsic colloidal aggregate state even increasing EDOT content. After post-treatment, conductivity improved dramatically, reaching a maximum at 10 wt% EDOT, followed by a decline to 183.94 S m^−1^ at 15 wt%. The pronounced enhancement (nearly 50-fold) still underscores the pivotal role of post-treatment in improving highly conductive PEDOT-rich domains, a process further supported by increased surface hydrophobicity (Fig. S13). Notably, the excessive monomer may lead to PEDOT:PSS homogeneity, thereby undermining its electron transport ability. A clear demonstration of the synergistic effect of the processing steps is shown in Fig. 3g, highlighting that each step (*i.e.,* vapor assist, ANF, DMSO/H_2_O exchange) is indispensable in constructing and stabilizing the PEDOT-rich conductive network.

The comprehensive advantage of our PAP hydrogel is further corroborated by a comparative analysis with previously reported PVA/PEDOT:PSS-based systems. As illustrated in Table S1 and visualized in Fig. 3h, our hydrogel distinctly occupies the upper right quadrant, concurrently achieving high conductivity and strength, well surpassing the conventional performance envelope of existing counterparts. These results underscore the efficacy of our synergistic strategy in the fabrication of conductive hydrogels with a well-balanced integration of mechanical robustness and electron transport capabilities.

### Biocompatibility of the PAP Hydrogel

**Fig. 4:**
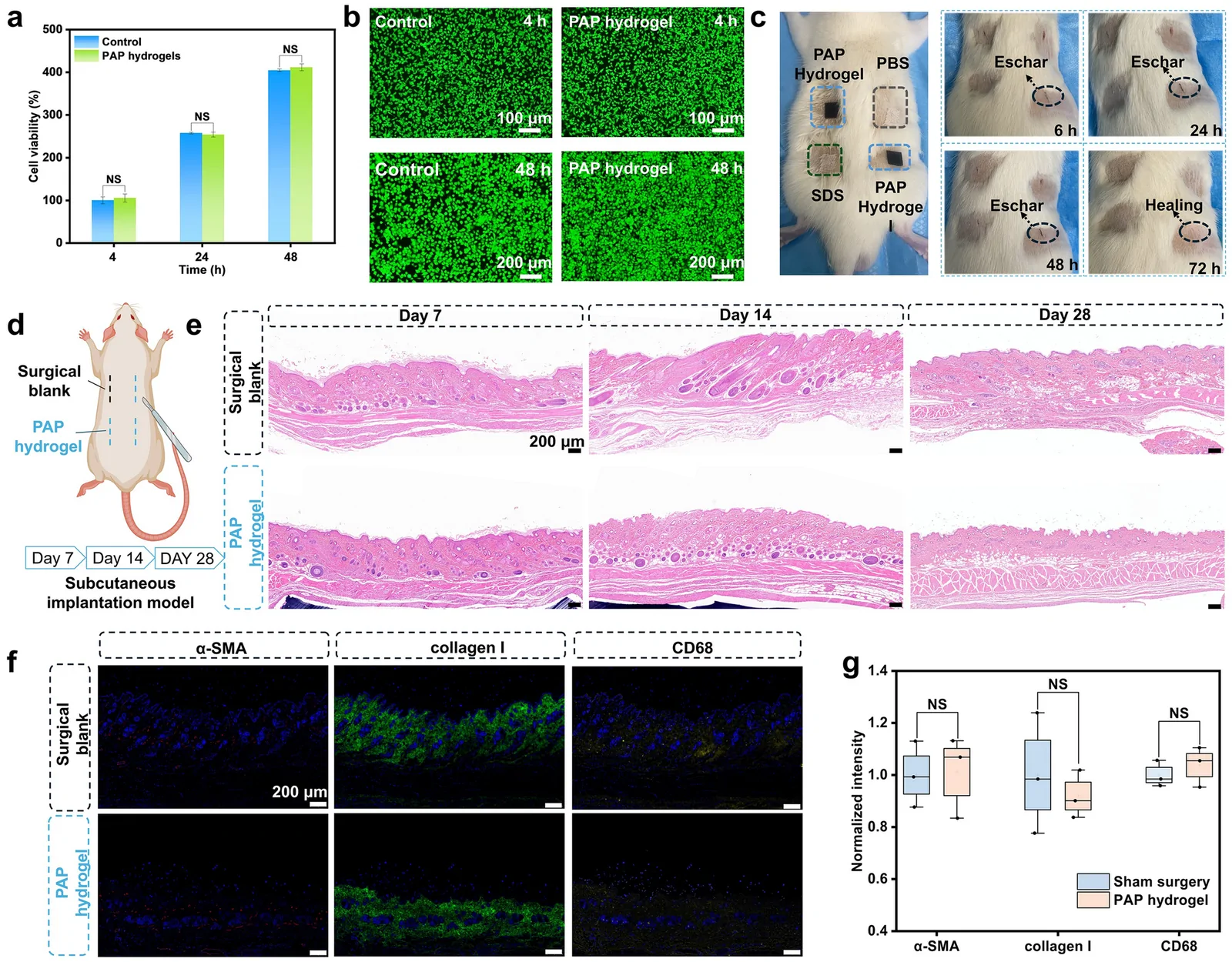
Biocompatibility assessment of the PAP hydrogel. **a** Cell viability of L929 fibroblasts after incubation with PAP hydrogel extract for 4, 24, and 48 h, as determined by the CCK-8 assay. **b** LIVE/DEAD staining images of L929 cells after incubation with the extract at 4 and 48 h.** c** Photographs of a rat skin irritation model showing the skin response at different time points (6–72 h) after topical exposure to PAP hydrogel, PBS, and SDS. **d** Schematic illustration of the subcutaneous implantation model in rats. **e** Hematoxylin and eosin (H&E) staining of tissue sections at the implantation site on days 7, 14, and 28 post-implantations. **f** Immunofluorescence staining and **g** quantification of normalized intensity of α-SMA, collagen I, and CD68 on day 28

Biocompatibility is a fundamental prerequisite for the successful development of hydrogels in wearable electronics. To this end, the PAP hydrogel was systematically assessed by L929 mouse fibroblasts. Although organic solvents such as n-hexane and DMSO were employed during synthesis, they were thoroughly eliminated via swelling and thermal annealing, thereby minimizing potential cytotoxicity. As presented in Fig. 4a, b, both CCK-8 assays and LIVE/DEAD staining demonstrated that the PAP hydrogel elicited no observable cytotoxicity on mouse embryonic fibroblast (MEF) cells, with cell viability and morphology closely resembling those of the control group at 4, 24, and 48 h. Additional hemocompatibility evaluations, including hemolysis and platelet adhesion tests, further confirmed the hydrogel’s blood compatibility, with a hemolysis rate below the 5% safety threshold and platelet attachment observed (Fig. S17).

Consistently, a skin irritation test revealed that the treated area remained intact with spontaneous recovery after 72 h of exposure to the PAP hydrogel, with no observable signs of erythema, edema, or eschar formation compared to the control group, confirming its non-irritant nature and compatibility with the natural healing process (Fig. 4c). Further subcutaneous implantation of the PAP gel into rats was conducted to examine tissue compatibility. As shown in Figs. 4d and S18, hydrogel samples and sham surgical blanks were implanted in symmetrical positions on the dorsal side. H&E staining of the surrounding tissue (Fig. 4e) showed no signs of inflammation, fibrotic encapsulation, or necrosis over 28 days, showcasing excellent histocompatibility and minimal foreign body response.

The host tissue response to the PAP hydrogel was evaluated via immunofluorescence staining of fibrosis- and inflammation-related markers, including *α*-SMA, collagen I, and CD68. As shown in Fig. 4f, g, the expression levels of these markers in the gel group were nearly indistinguishable from those in the surgical blank, indicating excellent in vivo biocompatibility with negligible fibrotic or inflammatory activation.

### Applications in Wearable Electronics

**Fig. 5:**
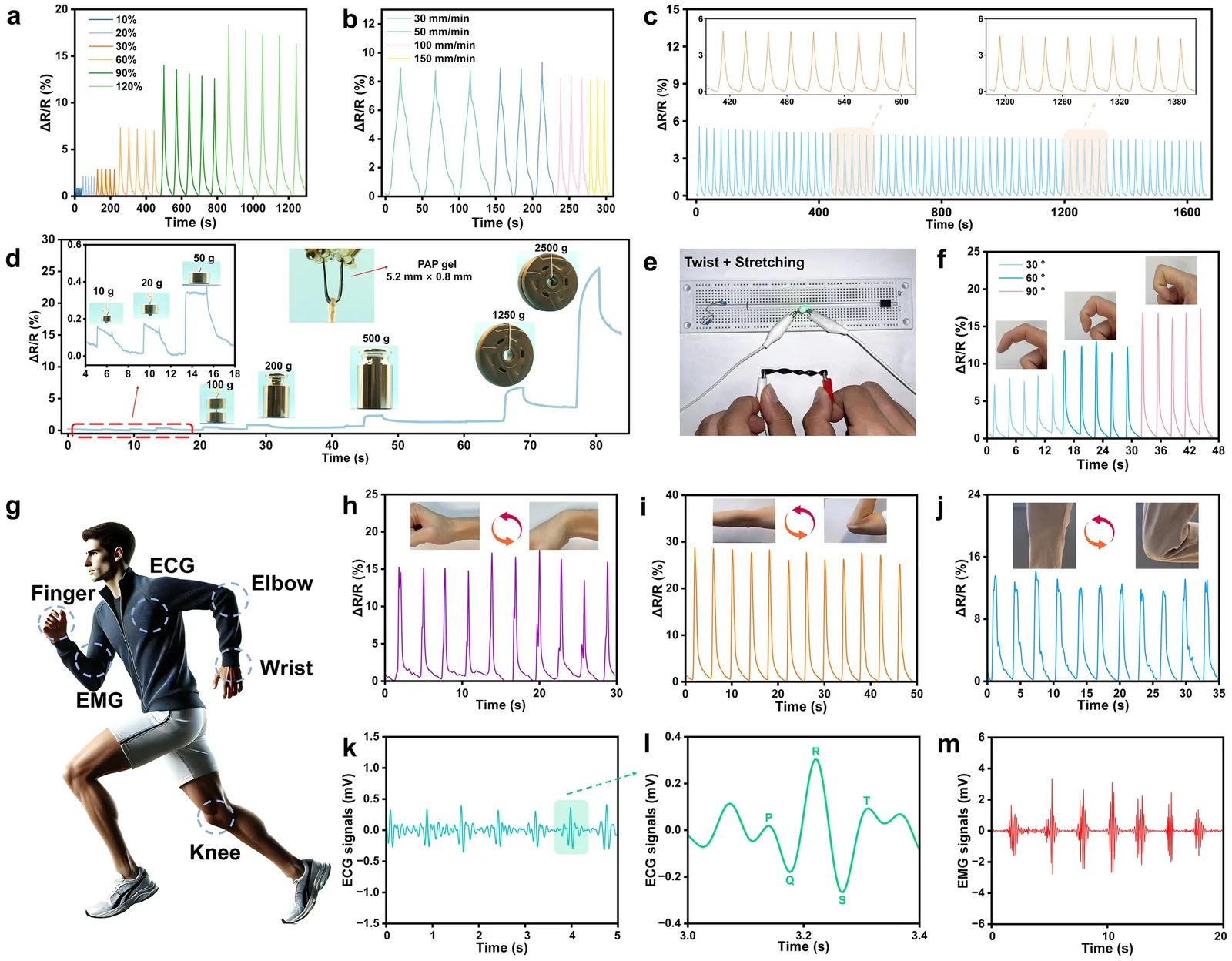
Strain sensing and bio-signal monitoring performance of the PAP hydrogel. Relative resistance variation under: **a** different strains and **b** various stretching rates. **c** Cyclic stability under repeated loading. **d** Detection of gradually increased weights up to 2.5 kg. **e** LED circuit demonstration under mechanical deformation. **f** Resistance variation during finger bending at different angles (30°, 60°, and 90°). **g** Schematic of sensor placement for human motion and electrophysiological signal monitoring. **h-j** Real-time resistance signals from wrist, elbow, and knee motions. **k** Real-time ECG signals recorded from the human body. **l** Enlarged ECG waveform showing characteristic P-Q-R-S-T peaks. **m** EMG signals collected during muscle activity

Based on the aforementioned properties, the PAP hydrogel was further evaluated as a multifunctional sensor for small to large strain sensing and electrophysiological signal monitoring. As illustrated in Fig. 5a, the sensor showed stable and repeatable relative resistance changes at various strain levels from 10% to 120%, enabling precise detection of both subtle and large-scale deformations. To assess the rate-dependent performance, the sensor was stretched at various speeds (Fig. 5b) and consistently delivered reliable responses across all tested rates. Furthermore, the sensor demonstrated a rapid response time of about 200 ms (Fig. S19a), ensuring timely signal output for real-time monitoring. Strain sensitivity was quantitatively assessed by calculating the gauge factor (GF), which increased from 0.32 (0–50%) to 1.42 (50%–100%) and 2.75 (100%–200%), respectively (Fig. S19b), revealing favorable sensitivity across a wide strain range. Notably, comparison with a purely geometric model reveals a pronounced deviation in the resistance–strain relationship, indicating that the sensing behavior is not governed solely by macroscopic-dimensional changes but instead reflects a deformation coupled electrical response, consistent with a functional analogy between mechanical and conductive networks in biological vascular–neural systems (Fig. S20) [7]. Long-term operational stability was verified through continuous cyclic stretching at 30% strain for 1600 s (Fig. 5c), and stable sensing performance was also maintained at higher frequencies (Fig. S21). The signal remained highly stable, highlighting the hydrogel’s mechanical durability and electrical reliability under dynamic conditions.

The PAP hydrogels couple high load-bearing capability with deformation-insensitive conduction under demanding perturbations. Gradual loading on a tiny PAP hydrogel (5.2 mm × 0.8 mm) generated distinct stepwise signals from gram- to kilogram-level weights and sustained up to 2.5 kg without mechanical or electrical failure (Fig. 5d), whereas a PVA-PEDOT:PSS control fractured at 100 g (Fig. S22). When integrated into a simple LED circuit, the LED remained steadily illuminated during both stretching and twisting (Fig. 5e). Under repeated hammering, the PAP-connected circuit still emitted steadily after 1 min of high-force, high-frequency blows, while the counterpart weakened and was damaged under low-force, lower-frequency hammering (Fig. S23). These results substantiate that the PAP hydrogel combines high mechanical strength with high conductivity, making it suitable for practical wearable applications.

The PAP hydrogel was also utilized in major joints, including the finger, wrist, elbow, and knee to enable comprehensive human motion monitoring (Fig. 5g). As shown in Fig. 5f, the sensor produced distinct and highly reproducible resistance responses at varying finger bending angles (30°, 60°, and 90°), with signal amplitude scaling proportionally with the degree of deformation. It also maintained reliable performance in capturing bending and stretching motions at other joints, including the elbow, wrist, and knee (Fig. 5h-j), further validating its effectiveness in diverse motion monitoring scenarios.

The PAP hydrogel was also mounted on the forearms and ankle to enable high-fidelity acquisition of ECG and EMG signals, further demonstrating its versatility as a multimodal sensing platform. For ECG recording, hydrogel sensors were positioned on the left and right forearms and a single ankle of a volunteer to form a stable and low-noise signal loop. As shown in Fig. 5k, the real-time ECG signals displayed clearly defined and stable waveforms, while the magnified segment (Fig. 5l) presented the characteristic P, Q, R, S, and T waves. In addition, the sensor was employed to monitor EMG signals during voluntary muscle activation. As demonstrated in Fig. 5m, when the volunteer repeatedly clenched and released the fist, distinct and periodic bursts with peak amplitudes of ~ 5–6 mV were observed, indicating strong neuromuscular activity and excellent signal fidelity under dynamic conditions. Stable EMG signals were reproducibly obtained over three consecutive days using the same electrodes (Fig. S24). Together, these findings demonstrate the capability of the PAP hydrogel to serve as a unified platform for multimodal sensing, enabling both strain and electrophysiological signal monitoring in wearable healthcare applications.

**Fig. 6:**
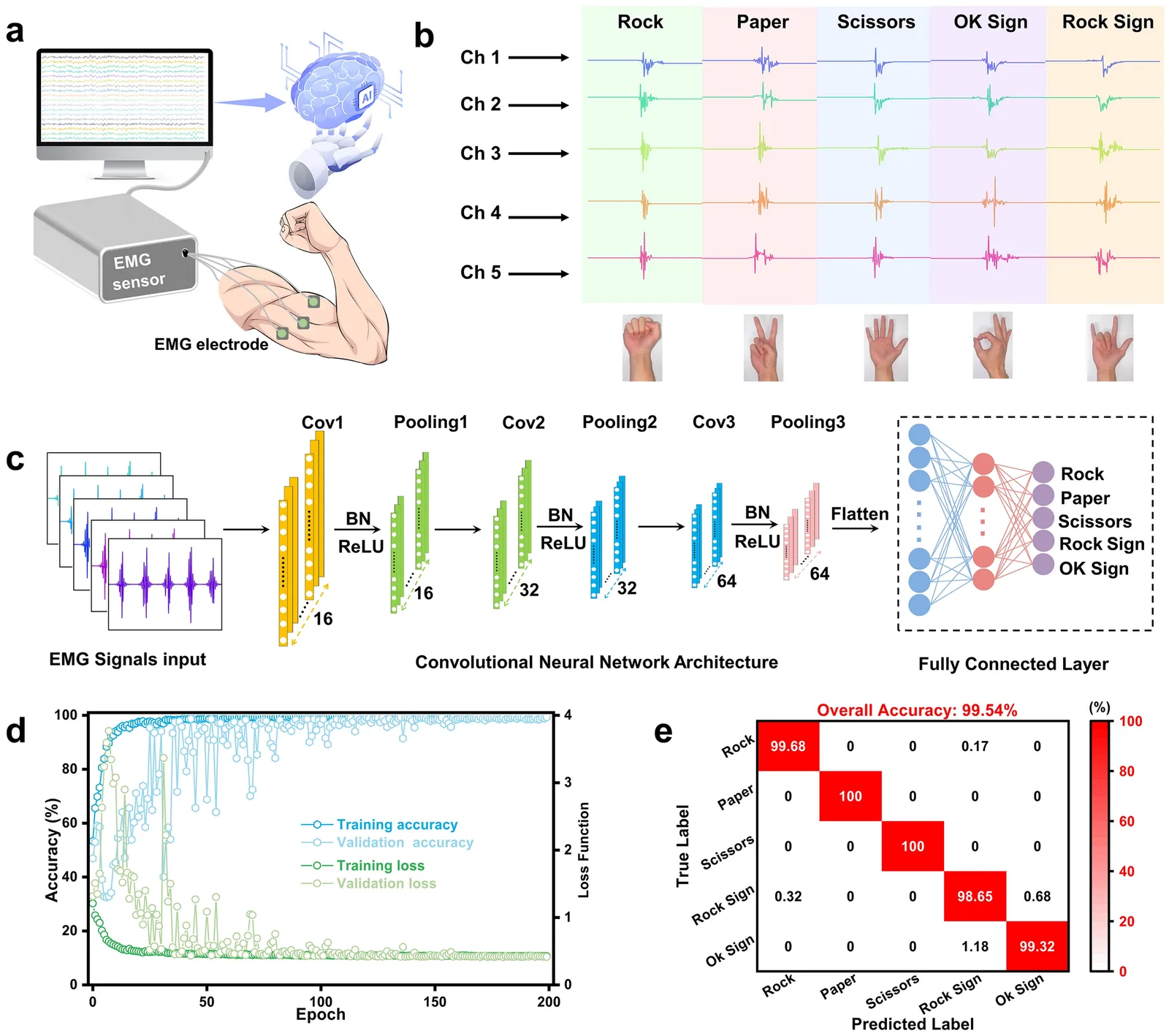
Design and performance of the EMG-based gesture recognition system. **a** EMG acquisition setup using hydrogel electrodes. **b** EMG signal patterns from five channels correspond to five different hand gestures. **c** CNN for gesture classification. **d** Training and validation accuracy and loss over 200 epochs. **e** Confusion matrix showing classification results with an overall accuracy of 99.54%

Machine learning, as a powerful branch of artificial intelligence, offers efficient and accurate solutions to decode various human motions. In particular, EMG-based gesture recognition has gained increasing attraction for applications in prosthetic control, rehabilitation, and sign language interpretation. By integrating hydrogel-based multi-channel EMG sensing with a tailored CNN, real-time and high-accuracy recognition of complex hand gestures was successfully demonstrated (Fig. 6). As illustrated in Fig. 6a, a total of seven hydrogel electrodes (five measurement electrodes, one shared reference, and one ground) were placed on the forearm to acquire five-channel EMG signals. The wearable system transmits the collected EMG signals wirelessly to a computer for real-time analysis and classification. The EMG patterns corresponding to five different sign gestures (rock, paper, scissors, rock sign and OK sign) exhibited distinct and recognizable signal amplitudes across the five channels (Fig. 6b), enabling reliable feature extraction for downstream classification.

A CNN architecture was designed to process and classify the EMG signals collected by the hydrogel sensor system (Fig. 6c). As shown in Fig. S25, the dataset was preprocessed and divided into training (60%), validation (20%), and testing (20%) subsets. The network consisted of three convolutional layers, each followed by batch normalization and ReLU activation, and terminated with a fully connected output layer for gesture classification. Model performance was assessed using accuracy and loss curves, which demonstrated rapid convergence and stable generalization over 200 training epochs (Fig. 6d). The resulting confusion matrix (Fig. 6e) reveals an overall recognition accuracy of 99.54%, validating the precision of the sensing and learning system in gesture recognition tasks. These results demonstrate the feasibility of PAP hydrogels as reliable EMG interfaces for multi-channel gesture recognition.

## Conclusions

Inspired by the cooperative vascular–neural networks in biological tissues, we developed a robust and highly conductive PAP hydrogel composed of PVA, ANF, and in situ polymerized PEDOT:PSS, effectively overcoming the long-standing mechanical–electrical trade-off in conductive hydrogels. The ANF-reinforced PVA network mimics vascular structures, providing mechanical support, while the PEDOT pathways resemble neural circuits, enabling efficient electron transport. The resulting bi-continuous architecture integrates mechanical and conductive networks, achieving a rare synergy of high tensile strength (10.72 MPa) and ultrahigh electrical conductivity (452.75 S m^−1^) with excellent biocompatibility, outperforming most reported PVA-PEDOT:PSS systems. The hydrogel maintained stable conduction under impact and complex deformation, supporting both stable joint motion tracking and high-fidelity acquisition of ECG and EMG signals. When integrated into a five-channel EMG system coupled with a custom CNN, it enabled accurate recognition of complex hand gestures with 99.54% accuracy, highlighting its strong potential for intelligent wearable electronics. This bioinspired strategy provides a general framework for designing next-generation intelligent wearable and biointegrated electronics.

## Supplementary Information

Below is the link to the electronic supplementary material.

Supplementary file1 (DOCX 10708 KB)
