# Supplementary material for: Bioinspired Structural Design Enables Synergistic Toughness and Conductivity in Hydrogels for Advanced Wearable Electronics
Source: Nanomicro Lett. 2026 Feb 9;18:249. doi: 10.1007/s40820-026-02094-y (PMC12886709; doi:10.1007/s40820-026-02094-y)
Supplement: Supplementary file 1 — Supplementary file1 (DOCX 10708 KB) [file 40820_2026_2094_MOESM1_ESM.docx]

Supporting Information for

**Bioinspired Structural Design Enables Synergistic Toughness and Conductivity in Hydrogels for Advanced Wearable Electronics**

Yi Liu^1^, Xuchen Wang^1^, Junjie Wang^1^, Zhuang Li^1^, Kelong Ao^1^, Guangwei Liang^1^, Haiqing Liu^1,2^, Qirui Zhang^1^, Mengjiao Pan^1^, Dahua Shou^1,3,4,5^ ⃰

^1^ Future Intelligent Wear Centre, School of Fashion and Textiles, The Hong Kong Polytechnic University, Hung Hom, Kowloon, Hong Kong, P. R. China

^2^ School of Intelligent Manufacturing and Smart Transportation, Suzhou City University, Suzhou 215104, Jiangsu, P. R. China

^3^ Research Centre of Textiles for Future Fashion, The Hong Kong Polytechnic University, Hung Hom, Kowloon, Hong Kong, P. R. China

^4^ Research Institute for Intelligent Wearable Systems, The Hong Kong Polytechnic University, Hung Hom, Kowloon, Hong Kong, P. R. China

^5^ PolyU-Xingguo Technology and Innovation Research Institute, The Hong Kong Polytechnic University, Hung Hom, Kowloon, Hong Kong, P. R. China

*Corresponding author. E-mail: [dahua.shou@polyu.edu.hk](mailto:dahua.shou@polyu.edu.hk) (Dahua Shou)

**S1 Characterizations**

**S1.1 Attenuated total reflection infrared spectroscopy (ATR-FTIR)**

FTIR spectra were recorded using a Spectrum 100 spectrometer (PerkinElmer, USA) equipped with an attenuated total reflectance (ATR). The wavenumber range was 4000-500 cm^-1^ with a resolution of 4 cm^-1^. Hydrogel samples were freeze-dried prior to measurement.

**S1.2 X-ray diffraction (XRD)**

XRD analysis was carried out by a Rigaku SmartLab X-ray diffractometer (9 kW rotating anode, Cu Kα radiation, *λ* = 1.5406 Å). Diffraction patterns were recorded in the range of 10^o^-50^o^ (2θ) with a step size of 0.02^o^, and scanning speed is 2^o^·min^-1^.

**S1.3 Wide- and small-angle X-ray scattering (WAXS/SAXS):**

WAXS measurements were conducted using an Anton Paar SAXSpoint 2.0 system (Anton Paar, Austria) equipped with a Cu Kα radiation source (*λ* = 0.154184 nm) operated at 40 kV and 50 mA. A two-dimensional hybrid photon counting detector (EIGER R 1M, Dectris; pixel size 75 µm) was used to collect the scattering patterns, and the sample-to-detector distance was set at 542 mm. The WAXS profiles were plotted as scattering intensity versus the scattering vector *q*, covering a *q* range of 5-30 nm⁻¹. The average size of crystalline domains (*D*) was estimated using the Scherrer equation:

$D=k\lambda/(\beta cos\theta)$ (S1)

where *k* is the Scherrer constant (typically taken as 1), *λ* is the X-ray wavelength, *θ* is the Bragg angle, and *β* is the full width at half maximum (FWHM) of the diffraction peak.

SAXS measurements were performed at the Shanghai Synchrotron Radiation Facility (SSRF), Shanghai, China. The X-ray beam energy was 12.0 keV (*λ* = 1.033 Å), and the sample-to-detector distance was 5713.0 mm. The detector pixel size was 172 µm × 172 µm, and silver behenate (AgBh) was used as the calibration standard. Hydrogel samples were measured at room temperature, and background scattering was recorded under the same conditions without the sample. The 2D SAXS patterns were integrated into 1D scattering profiles using Fit2D software, and data were corrected for beam fluctuation, detector distortion, and background noise. The effective scattering vector (*q*) range was 0.003-0.2 nm^-1^. To identify structural periodicity, the product of scattering intensity (*I*) and *q^2^* was plotted against *q*. The average interdomain spacing (*L*) was calculated using Bragg’s equation:

$L=2\pi/q_{max}$ (S2)

Where *q_max_* is the scattering vector corresponding to the maximum peak intensity.

*Scanning electron microscopy (SEM):* Freeze-fractured and freeze-dried hydrogel cross-sections were sputter-coated with gold and imaged using a Hitachi S-4800 FE-SEM (Japan).

**S1.4 Raman spectroscopy**

Raman spectra were obtained using a HORIBA LabRAM HR Evolution spectrometer with a 532 nm laser, over the range 1000–1800 cm^-1^.

*X-ray photoelectron spectroscopy (XPS):* XPS was conducted using a Thermo Scientific ESCALAB QXi spectrometer equipped with a monochromatic Al Kα X-ray source. The sulfur (S 2p) signals were analyzed to determine the relative content of PEDOT and PSS. Prior to in-depth analysis, ion etching was applied for 30 s to investigate near-surface composition. The binding energies were calibrated using the C 1s peak at 284.8 eV as the reference.

**S1.5 Atomic force microscopy (AFM)**

AFM was performed using an Asylum Research MFP-3D atomic force microscope in tapping mode at 256 × 256 pixels with a scan rate of 1 Hz.

**S1.6 Mechanical properties**

Tensile tests were performed on a universal testing machine (Instron 5566, Instron USA) equipped with a 500 N load cell at a strain rate of 100 mm min^-1^ under 20 ℃ ± 2 ℃ and 65% RH. The samples were cut into rectangles, and their length, width, and thickness were measured using a vernier caliper prior to testing. Young's modulus *E*, fracture stress *σ_b_*, and work of extension *W*, were determined from uniaxial stress-strain curves. *E* was calculated based on the initial slope of the stress-strain curve within a strain range below 10%, while *W* was determined by integrating the area under load-extension curves. For cyclic tests, silicone oil was used to surfaces of the gels to prevent water loss. Repeated loading-unloading was performed under fixed or progressively increasing strain to evaluate mechanical durability and energy dissipation. All hydrogel samples were equilibrated in DI water for at least 2 days. Prior to measurements, samples were gently blotted to remove surface water and tested immediately under ambient conditions. At least three parallel samples were tested for each hydrogel formulation, and average values were reported.

**S1.7 Measurement of water content**

The water content of the hydrogels was measured using a halogen moisture analyzer (Furbs, FK-20A, China). Measurements were conducted at 130 °C for at least 20 min until a stable reading was obtained. For each formulation, at least three specimens were tested, and the average value was reported.

**S1.8 Conductivity properties**

The electrical conductivity (*σ*) of the hydrogels was determined by measuring the resistance (*R*) using both a digital multimeter (a two-probe method) and a four-probe method to exclude potential contact resistance effects. In both configurations, electrical contacts were prepared by inserting/anchoring metallic wires onto the hydrogel and fixing them with silver paste, providing a stable contact area. For the two-probe measurement, the resistance was recorded between the two outer electrodes. For the four-probe measurement, a constant current was applied through the two outer electrodes, and the voltage drop was collected between the two inner electrodes. The conductivity was calculated according to the following equation:

$\sigma=L/(RA)$ (S3)

where *σ* is the electrical conductivity (S m^-1^), *L* is the distance between the two electrodes (two-probe) or between the two inner voltage sensing electrodes (four-terminal) (m), *R* is the measured resistance (Ω), and *A* is the cross-sectional area of the sample (*A* = *w* × *d*, in m^2^).

Electrochemical impedance spectroscopy (EIS) and DC polarization measurements were performed using an electrochemical workstation (CHI 760E, CH Instruments) at room temperature in a two-electrode configuration. The hydrogel sample was measured in ambient air and was clamped between two platinum (Pt) electrodes with an effective contact area of ~5 × 9 mm^2^. A thin layer of silver paste was applied at both hydrogel/Pt interfaces to reduce interfacial contact resistance. EIS was carried out with a 5 mV AC perturbation at 0 V DC bias over a frequency range of 100 kHz to 0.1 Hz (quiet time: 2 s, one cycle per frequency). DC polarization was evaluated by chronoamperometry using a step potential from 0 to 0.05 V with a pulse width of 300 s (sampling interval: 0.1 s, quiet time: 5 s).

**S1.9 Sensing performance test**

The sensing performance of the hydrogel-based strain sensor was evaluated by monitoring the resistance change under mechanical deformation. The hydrogel was cut into rectangular strips, and silver paste was used to attach copper wires at both ends as electrodes. VHB tapes were applied to the top and bottom surfaces of the hydrogel to ensure firm contact and mechanical stability. The resistance was measured in real time using a high-precision digital multimeter (Keysight 34470A, 7½ Digit, Keysight Technologies, USA), while the sample was subjected to cyclic stretching or different strain levels. The relative resistance change was calculated using the following formula:

$\frac{\Delta R}{R}=\frac{(R-R_{0})}{R_{0}}$ (S4)

where *R₀* and *R* are the initial resistance and the resistance under deformation, respectively.

The strain sensitivity was quantified by the gauge factor (*GF*), calculated as:

$GF=\left( \frac{\Delta R}{R_{0}\cdot\varepsilon} \right)\times100\%$ (S5)

where *ε* is the applied tensile strain.

**S1.10 Biocompatibility assessment**

In vitro, L929 fibroblasts were seeded in 96-well plates at a density of 1 × 10^4^ cells/well and incubated for 24 h. Hydrogel extracts were prepared by incubating the samples in complete MEM medium (supplemented with 10% fetal bovine serum and 1% penicillin–streptomycin) at 0.1 g/mL for 72 h at 37 °C. After replacing the original medium with 100 μL of extract, cell viability was assessed at 4, 24, and 48 h using the CCK-8 assay. At each time point, 10 μL of CCK-8 solution was added to each well and incubated for 2 h, followed by measurement of absorbance at 450 nm. Cells cultured in fresh MEM complete medium were used as the blank control (100% viability). All experiments were conducted in triplicate, and results were reported as mean ± standard deviation (SD).

To evaluate the skin irritation potential of the hydrogel, square samples (approximately 1 cm × 1 cm) were applied to the shaved dorsal skin of SD rats (n = 8). The hair on the back was removed using an electric razor, followed by a 24 h recovery period. Each hydrogel sample was fixed to the skin using medical gauze and removed after 6 h of contact. Gauzes soaked with PBS (1×, GIBCO) and 20 wt% sodium dodecyl sulfate (SDS) were used as negative and positive controls, respectively. After the sample removal, the skin condition was visually examined and photographed at 6, 24, 48, and 72 h to monitor erythema, eschar formation, and healing.

The hemocompatibility of the PAP hydrogel was evaluated by a standard hemolysis assay using fresh blood from healthy SD rats, provided by the Experimental Animal Center of The Hong Kong Polytechnic University. Fresh blood was collected from healthy SD rats and anticoagulated with sodium citrate. Red blood cells were isolated by centrifugation at 3000 rpm for 10 min and washed three times with 0.9% NaCl. The hydrogel samples were incubated with 5 mL of diluted red blood cell suspension (2% in 0.9% NaCl) at 37 °C for 2 h. Deionized water and 0.9% NaCl were used as positive and negative controls, respectively. After incubation, all samples were centrifuged, and the absorbance of the supernatant was measured at 545 nm using a microplate reader. The hemolysis rate (H) was calculated using the following equation:

$H \left( \% \right)=\frac{{OD}_{s}-{OD}_{nc}}{{OD}_{pc}-{OD}_{nc}}\times100\%$ (S6)

where *OD_s_*​, *OD_nc_*​, and *OD_pc_*​ represent the absorbance of the sample, negative control, and positive control, respectively.

For platelet adhesion assay, platelet adhesion on the PAP hydrogel surface was assessed using platelet-rich plasma (PRP) collected from fresh rat blood. The blood was anticoagulated with sodium citrate and centrifuged at 700 rpm for 8 min at 8 °C to obtain PRP. Hydrogel samples were incubated in PRP at 37 °C for 2 h. After incubation, samples were gently washed with PBS to remove non-adherent platelets and fixed in 2.5% glutaraldehyde overnight at 4 °C. The samples were then dehydrated through a graded ethanol series, dried, and sputter-coated with gold for scanning electron microscopy (SEM) observation.

To evaluate the in vivo biocompatibility of the hydrogels, square-shaped samples (approximately 5 mm × 5 mm) were subcutaneously implanted into the dorsal region of SD rats under aseptic conditions. A sham-operated group receiving equal volumes of phosphate-buffered saline (PBS) served as the control. After 7 days, the animals were sacrificed, and the tissue surrounding the implantation site was carefully harvested for histological analysis. The excised tissues were fixed in 4% paraformaldehyde for 24 h at room temperature, followed by graded dehydration using ethanol solutions ranging from 75% to 100%, and subsequently cleared in xylene. The specimens were then embedded in paraffin, sectioned into 5 μm-thick slices, and stained with hematoxylin and eosin (H&E). Histological images were acquired using a digital whole-slide scanner (e.g., Panoramic MIDI, 3DHISTECH). All histological evaluations were performed in a blind manner without sample exclusion.

For immunofluorescent analysis, paraffin-embedded tissue sections were first deparaffinized, rehydrated with a graded ethanol series, and washed three times with PBS (5 min each). To block nonspecific binding, the slides were incubated with 5% goat serum for 20 min at room temperature. Primary antibodies were then applied and incubated overnight at 4 °C, including mouse anti-α-SMA (1:200, CST, 19245), rabbit anti-collagen I (1:500, Proteintech, 14695-1-AP), and mouse anti-CD68 (1:500, Abcam, ab125212) for fibroblasts, collagen, and macrophages, respectively. After primary incubation, the slides were rinsed in PBS and incubated for 30 min with Alexa Fluor 488–conjugated secondary antibodies (1:1000, Thermo Fisher). Nuclei were counterstained with DAPI (C1002, Beyotime) for 20 min. Fluorescent images were acquired using a confocal laser scanning microscope (SP8 STED 3X, Leica), and fluorescence intensities were quantified using ImageJ software. All image acquisition and analysis procedures were performed in a blinded manner without data exclusion.

**S1.11** **Electrophysiological signals acquisition and machine learning analysis**

For all electrophysiological signal recordings, hydrogel-based electrodes with dimensions of 1 cm × 1 cm and a thickness of approximately 1 mm were used. These hydrogel electrodes were non-adhesive and were gently fixed to the skin using medical adhesive tape to ensure stable contact during ECG and EMG recordings. All tests were conducted at room temperature to ensure material stability during measurements. The electrodes were connected to a biosensing platform (OpenBCI Cyton), and informed written consent was obtained from all participants for the recording of electrocardiogram (ECG) and electromyogram (EMG) signals.

For the long-term stability test, the same hydrogel electrodes were used for EMG recordings for 30 minutes per day over three consecutive days. After each recording session, the electrodes were rinsed and stored in deionized water to prevent dehydration. Before each subsequent measurement, the electrodes were simply taken out of the water and gently wiped to remove excess moisture before use.

For ECG measurements, an OpenBCI Cyton board was used to collect the signals. Two hydrogel electrodes spaced 3 cm apart were placed on the left arm, while the right arm and right ankle were used for reference and ground electrodes, respectively. For EMG recordings, a five-channel hydrogel electrode array was circumferentially attached around the forearm muscles of the right hand. Subjects were instructed to perform predefined hand gestures at 5-second intervals. The signals were recorded in real time using the OpenBCI Cyton system. The recorded signals were subsequently processed using MATLAB R2024b, including baseline drift removal, bandpass filtering (7-13 Hz), powerline interference suppression, and wavelet denoising to enhance signal quality for analysis.

**S1.12 Machine Learning for EMG-Based Gesture Classification**

EMG-based gesture recognition was conducted using a dataset comprising 60 signal recordings per gesture class, across eight distinct gesture types. Each signal was first standardized using z-score normalization and segmented into 5-second temporal windows. These segments were then categorized into five predefined gesture classes and randomly divided into training (60%), validation (20%), and test (20%) subsets. A compact convolutional neural network (CNN) was designed to extract temporal features hierarchically from the multichannel EMG signals. The model consisted of three one-dimensional convolutional layers with kernel size 5, stride 1, and padding 2, each followed by max-pooling layers (kernel size 2, stride 2). Temporal alignment of the input signals was preserved through symmetric padding. After convolution, the features were flattened and passed through two fully connected (FC) layers, with the first projecting into a 128-dimensional latent space and the second producing the final classification logits. ReLU functions were employed as activation units throughout, and a dropout layer with a rate of 0.5 was added between FC layers to alleviate overfitting.

The training process used the Adam optimizer (learning rate = 1×10^-4^) in conjunction with a cross-entropy loss function. Model performance was validated through stratified 5-fold cross-validation to assess its generalization capabilities. Training was conducted for up to 200 epochs with a batch size of 128, and early stopping was implemented based on stagnation of the validation loss. All experiments were performed using PyTorch 2.1.1 on a workstation equipped with an NVIDIA RTX 3080 GPU. Classification performance was quantified using both accuracy (ACC) and the weighted F1 score.

**S2 Supplementary Figures and Tables**


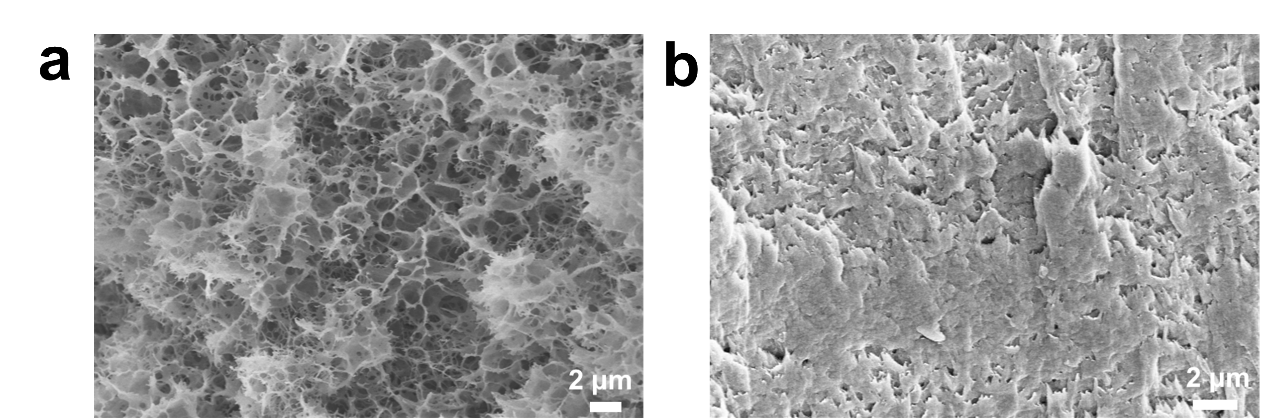


**Fig. S1** SEM images of freeze-dried hydrogels. **a** Porous network of PVA-ANF gel after freeze–thaw and water immersion. **b** Densified and aligned structure of PAP gel after PEDOT:PSS polymerization and annealing


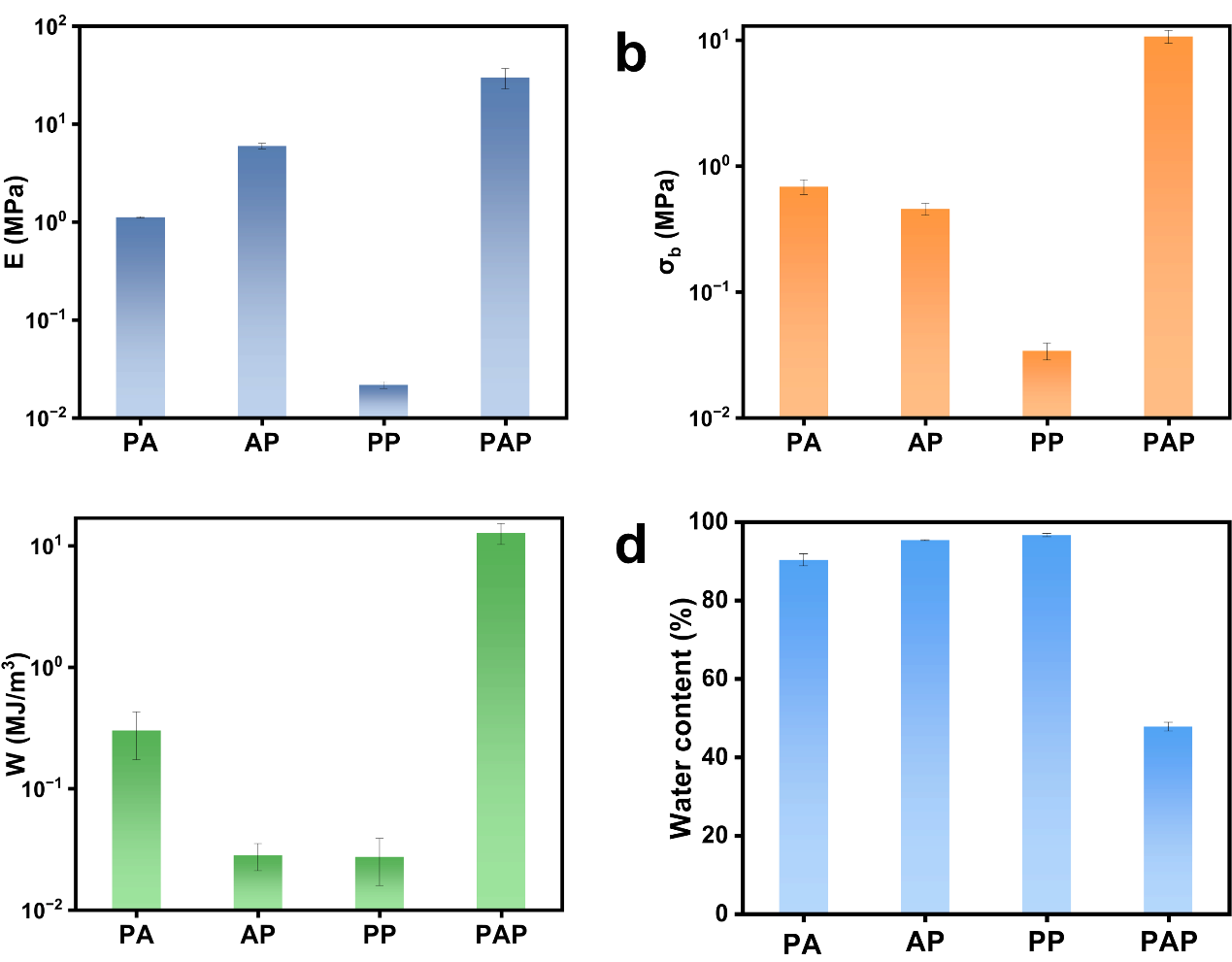


**Fig. S2** Mechanical properties and water content of different hydrogels


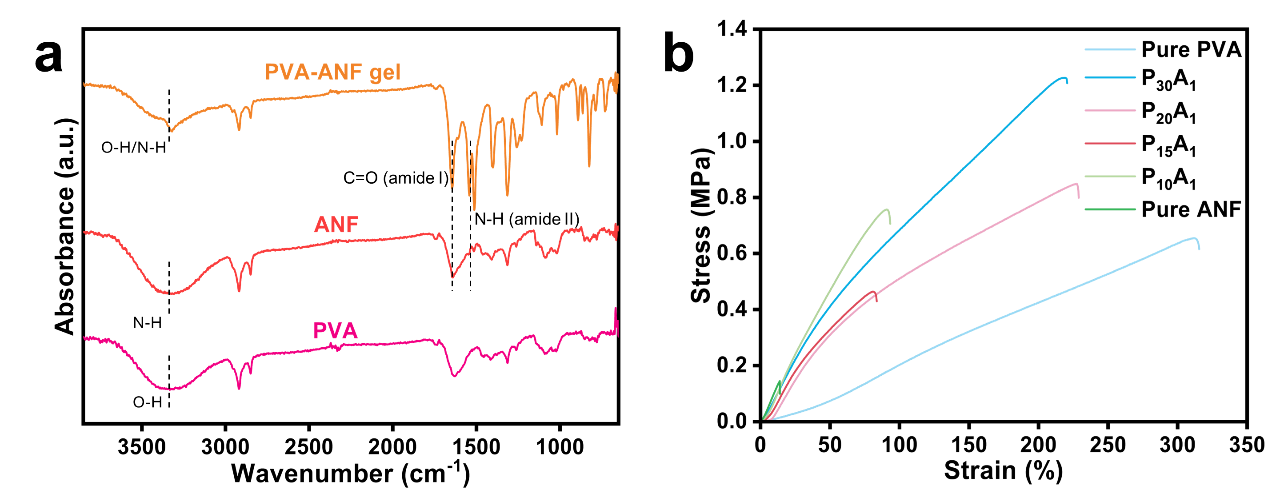


**Fig. S3** **a** FTIR spectra of PVA, ANF, and PVA-ANF gels. **b** Tensile stress-strain curves of pure PVA, pure ANF, and PVA-ANF hydrogels with various PVA-to-ANF mass ratios (P_x_A_1_). Increasing ANF content significantly improves the mechanical strength and modulus of the hydrogel, confirming the reinforcing effect of ANFs within the hydrogen-bonded polymer network


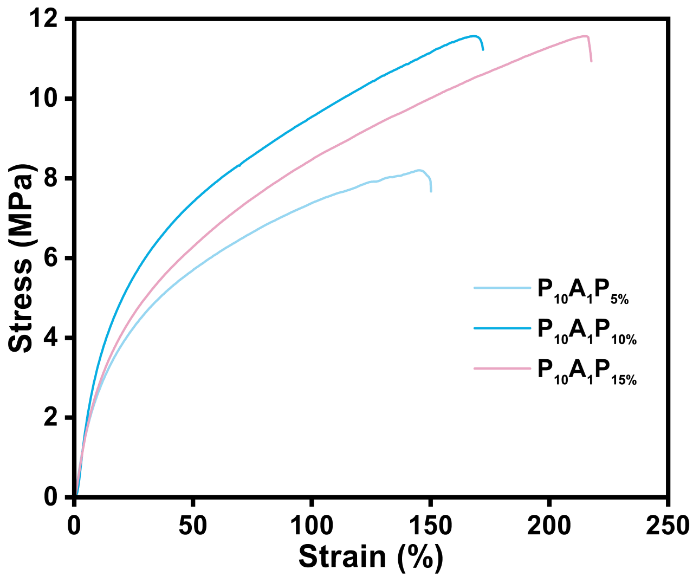


**Fig. S4** Tensile stress–strain curves of P₁₀A₁P hydrogels synthesized with varying EDOT concentrations (5-15 wt%)


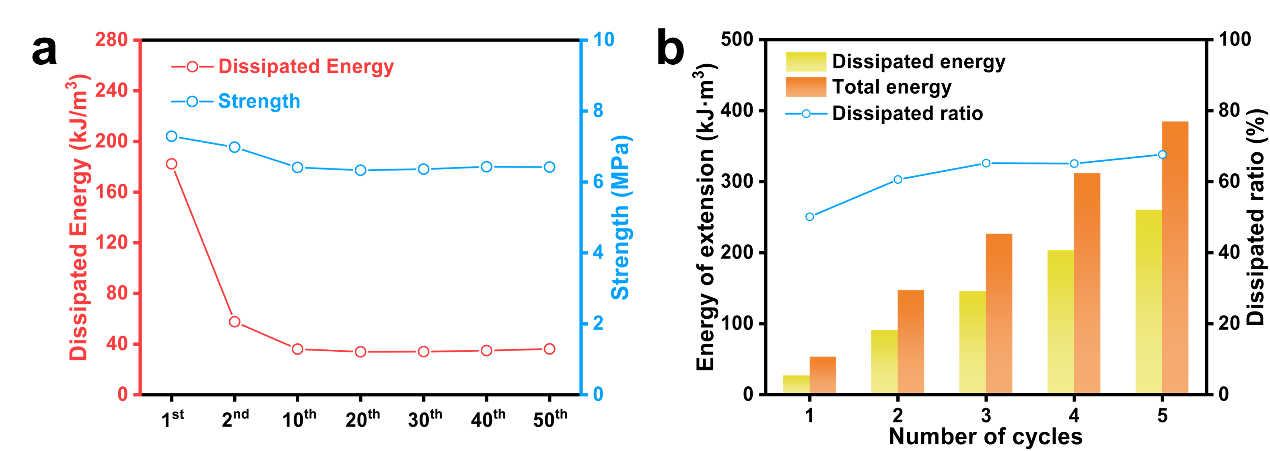


**Fig. S5** **a** Dissipated energy and strength of the P_10_A_1_P_10_ hydrogel during 50 cyclic stretching-releasing tests at a fixed strain of 50%. **b** Energy dissipation behavior of the P_10_A_1_P_10_ hydrogel under progressively increasing strain


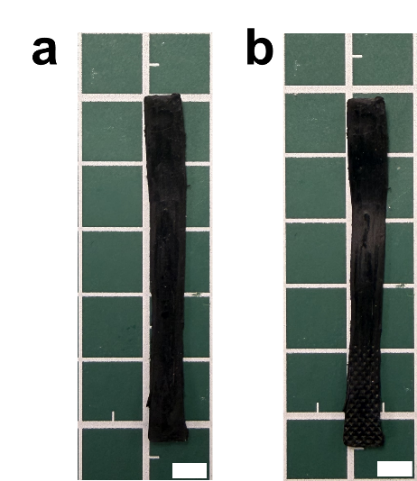


**Fig. S6** Digital photographs of the PAP hydrogel strip (a) before and (b) after 50 loading-unloading cycles at 50% strain (unloaded to 0% strain). The sample shows negligible permanent deformation after cyclic stretching. (scale bar = 50 mm)


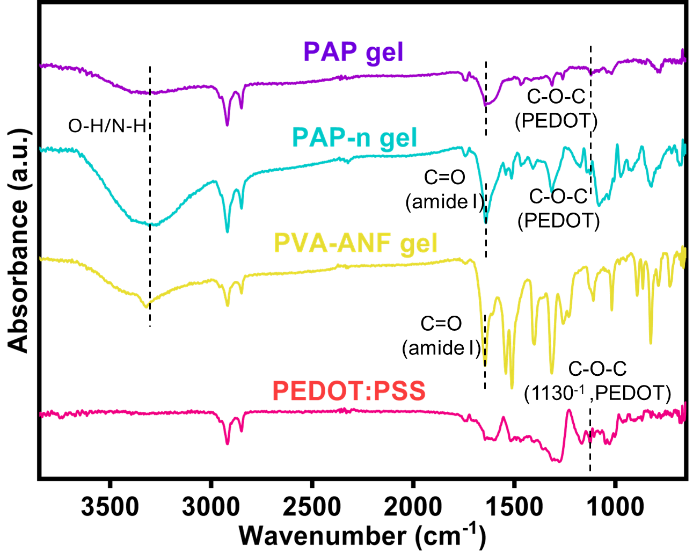


**Fig. S7** FTIR spectra of PEDOT:PSS, PVA-ANF gel, PAP-n gel (no annealed), and PAP gel (annealed). Characteristic peaks of PEDOT:PSS, including the C-O-C stretching vibration at ~1130 cm^-1^, appear in both PAP-n and PAP gels, confirming successful incorporation of the conductive polymer. The O-H/N-H stretching band in the PAP gel becomes broader and more intense compared to PAP-n, suggesting enhanced hydrogen bonding after annealing. These changes suggest improved interfacial interactions and better PEDOT chain ordering within the hydrogel matrix


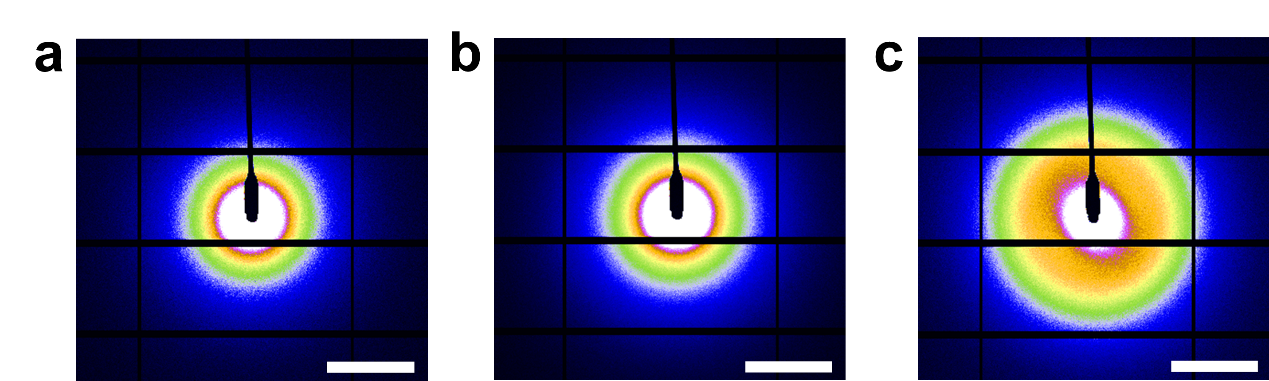


**Fig. S8** 2D SAXS patterns of PP **a** PAP-n, **b** and PAP **c** hydrogels. The enhanced scattering ring in the PAP sample indicates improved nanoscale structural ordering after annealing


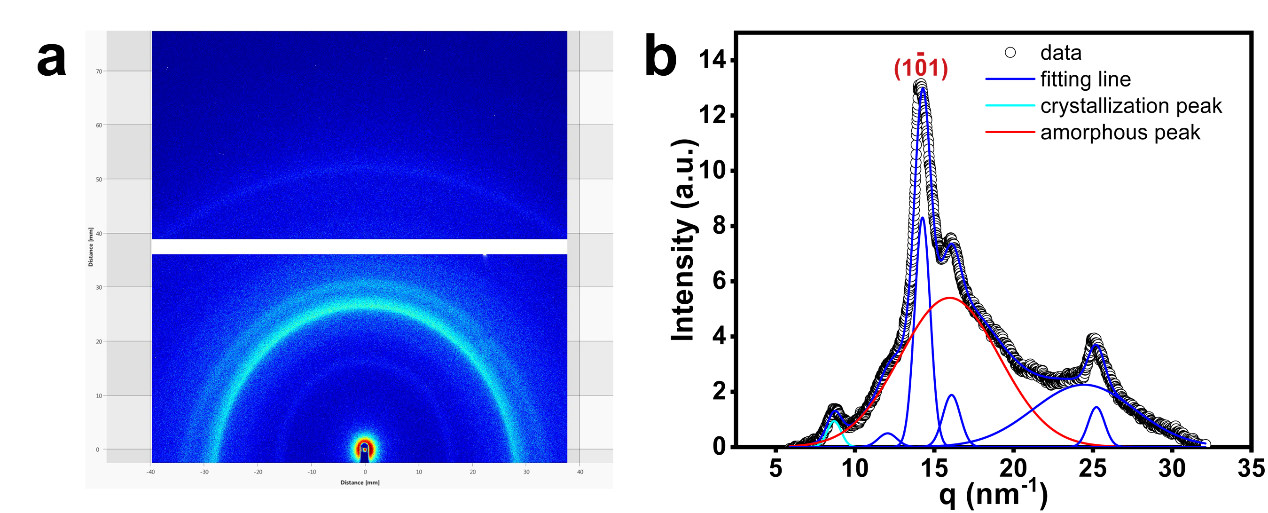


**Fig. S9 a** 2D WAXS pattern of the annealed PAP hydrogel, showing a well-defined semicrystalline diffraction ring. **b** Azimuthally integrated 1D WAXS profile and fitting of the PAP hydrogel. The sharp peak at q ~ 14.2 nm^-1^, assigned to the (101) crystalline plane of PVA, is separated from the amorphous background, confirming the coexistence of crystalline and amorphous domains


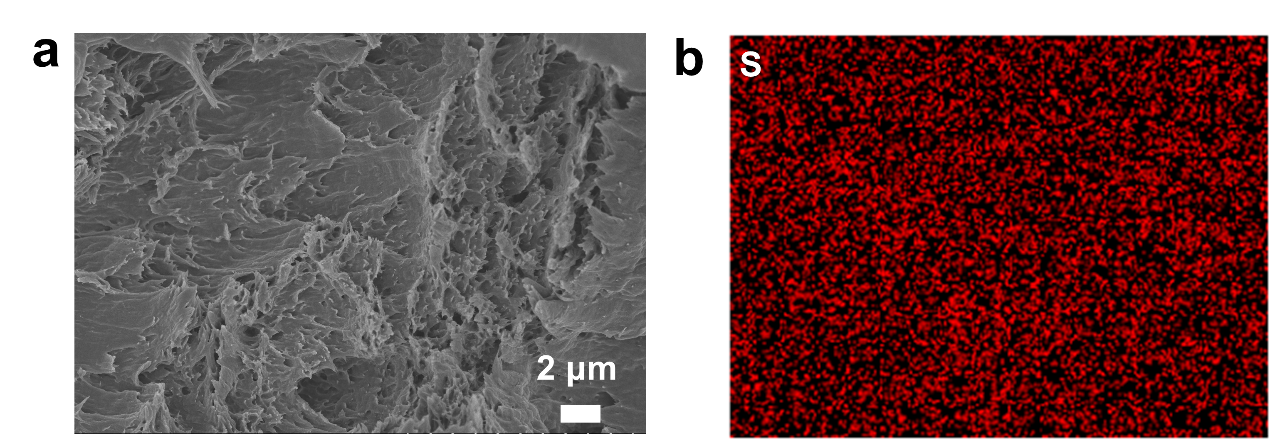


**Fig. S10** Cross-sectional SEM image and corresponding EDS sulfur mapping of the PAP hydrogel


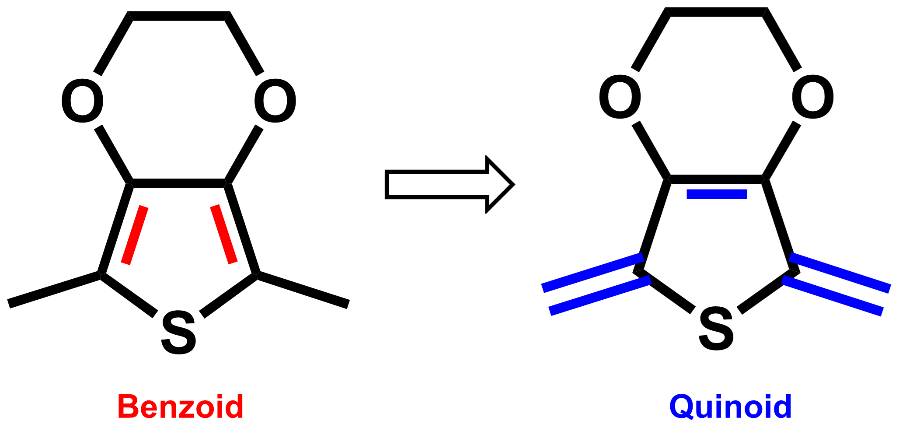


**Fig. S11** The chemical structure of PEDOT changes from benzoid structure to quinoid structure


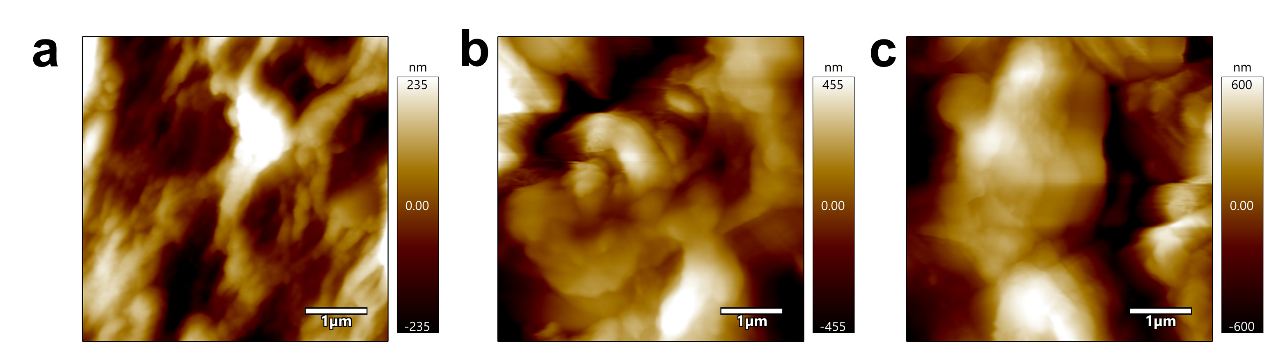


**Fig. S12** Atomic force microscopy phase images of **a** PEDOT:PSS; **b** PP hydrogel; **c** PAP hydrogel


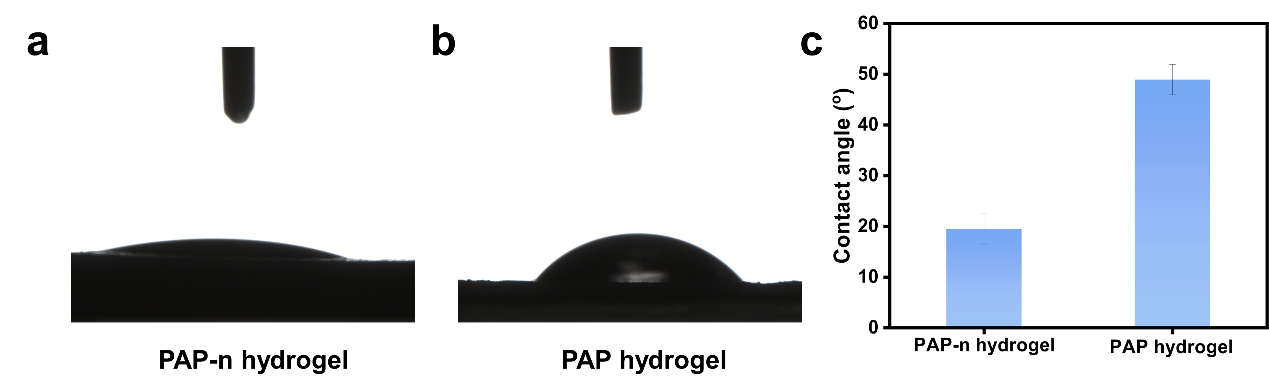


**Fig. S13** Water contact angle measurements of **a** PAP-n hydrogel and **b** PAP hydrogel. **c** Quantitative comparison of contact angles, indicating increased surface hydrophobicity after post-treatment


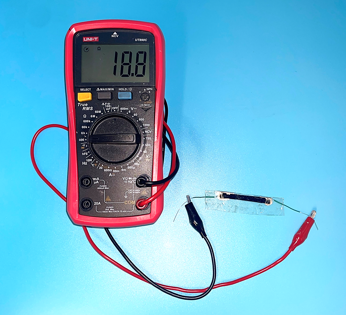


**Fig. S14** Photograph of a two-probe conductivity measurement of the PAP hydrogel using a digital multimeter. The hydrogel sample had a length of 48.52 mm, width of 5.17 mm, and thickness of 0.78 mm


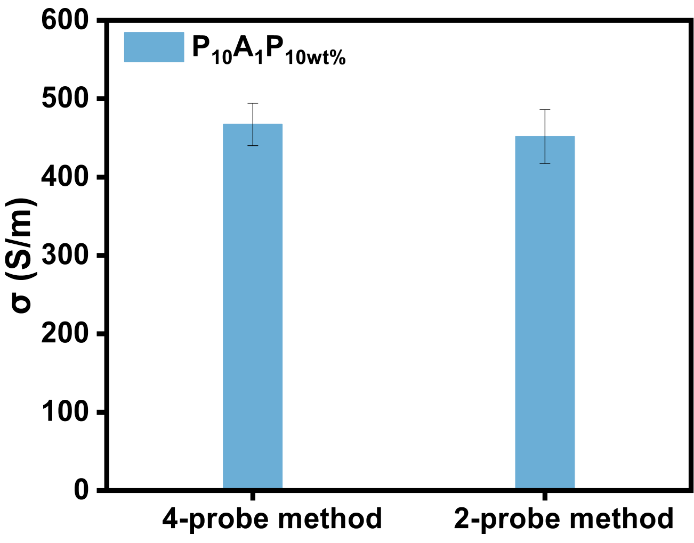


**Fig. S15** Comparison of electrical conductivity (σ) of the hydrogel measured by the four-probe method and the two-probe method. Data are presented as mean ± s.d. n = 3


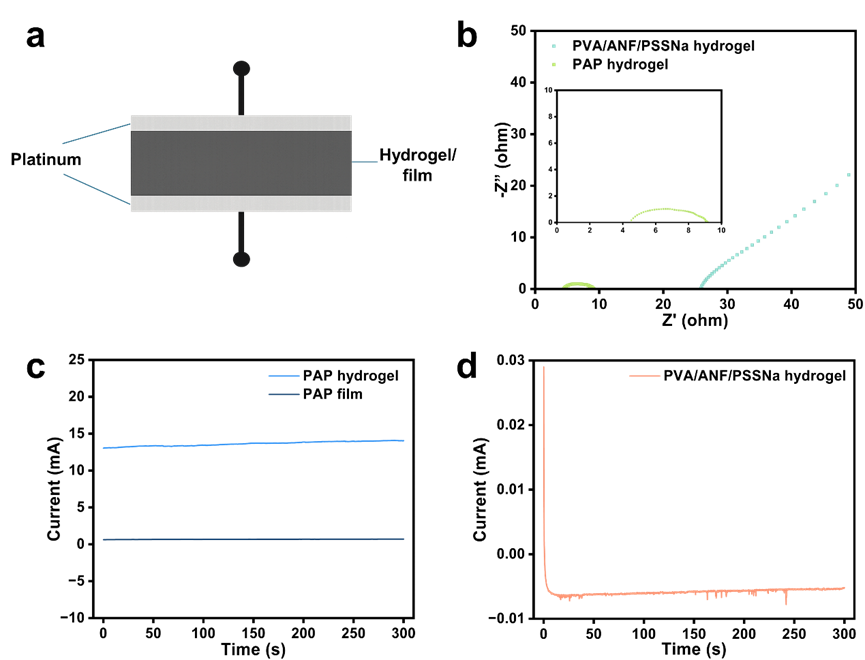


**Fig. S16** Charge-transport characterization of PAP hydrogels. (**a**) Schematic illustration of the sandwich-type electrode configuration. (**b**) Nyquist plots of the PAP hydrogel and PVA/ANF/PSSNa hydrogel measured by electrochemical impedance spectroscopy. (**c**) DC polarization curves of the PAP hydrogel, the dry PAP film and (**d**) PVA/ANF/PSSNa hydrogel under a constant bias


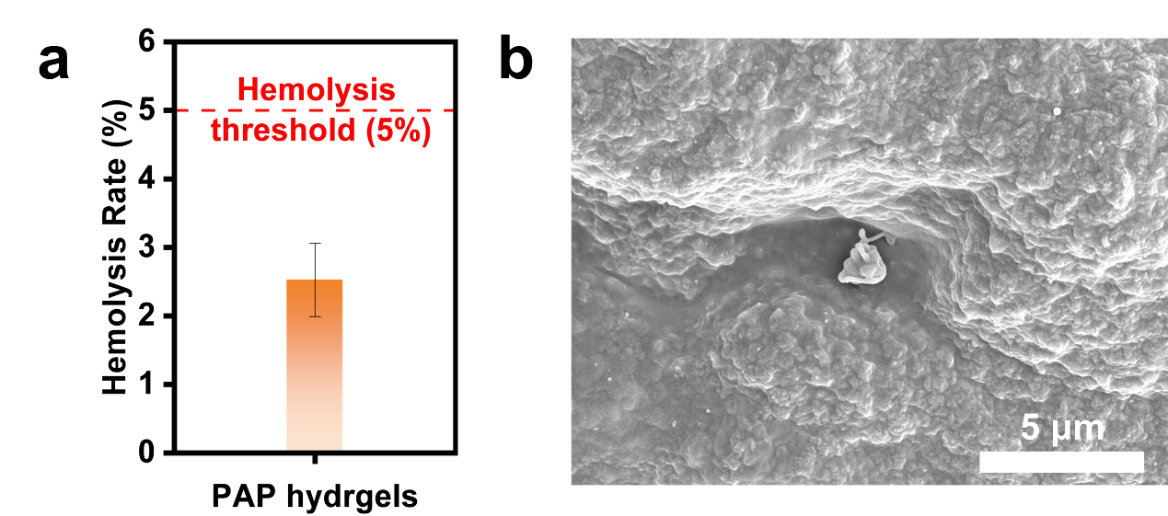


**Fig. S17** **a** Hemolysis test of the PAP hydrogel. **b** SEM image of platelet adhesion on the PAP hydrogel surface


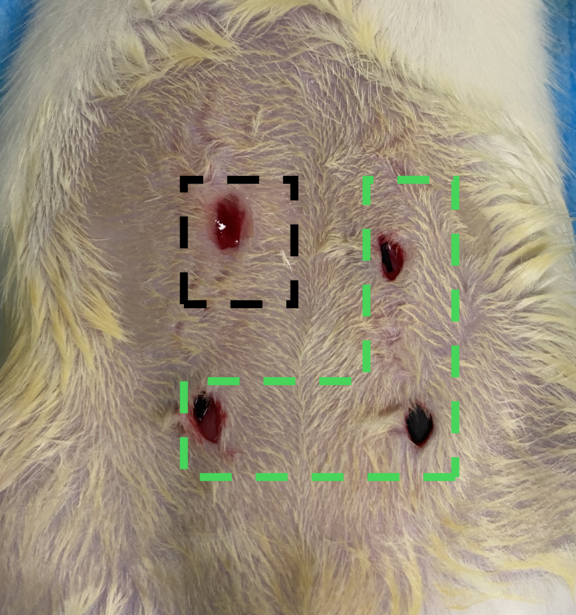


**Fig. S18** Photograph of subcutaneous implantation in rats for biocompatibility evaluation


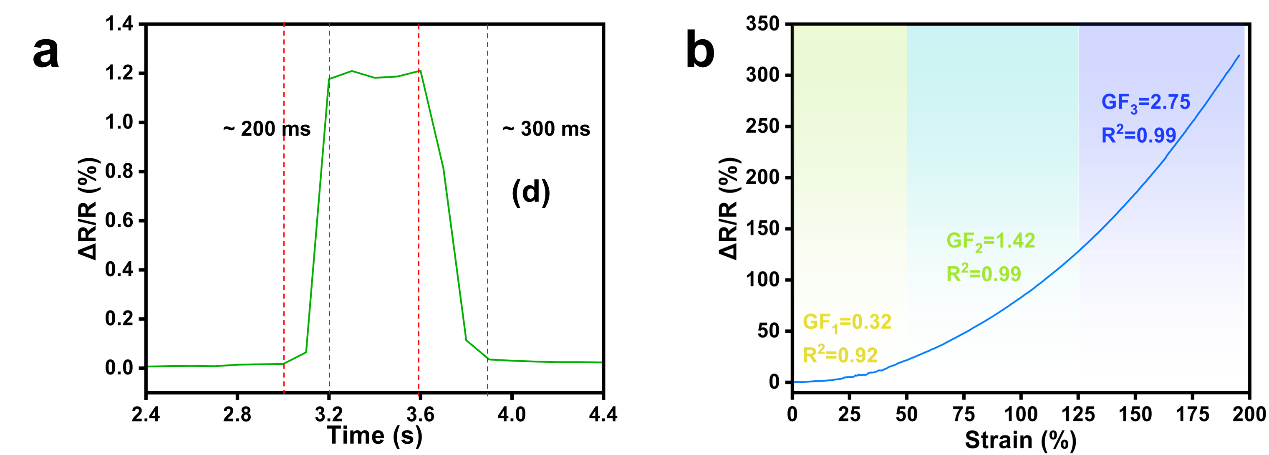


**Fig. S19 a** Response time of the PAP hydrogel sensor. **b** Gauge factor across varying strain ranges





**Fig. S20** Comparison between the experimentally measured resistance change and the purely geometric prediction as a function of tensile strain. The experimental $R/R_{0}$-strain curve (solid line) shows a pronounced deviation from the theoretical calculation $R/R_{0}=\lambda^{2}$(dashed line), which is based on the assumption constant resistivity and incompressible deformation (Poisson’s ratio$\nu\approx0.5$, $\lambda=1+\varepsilon$)


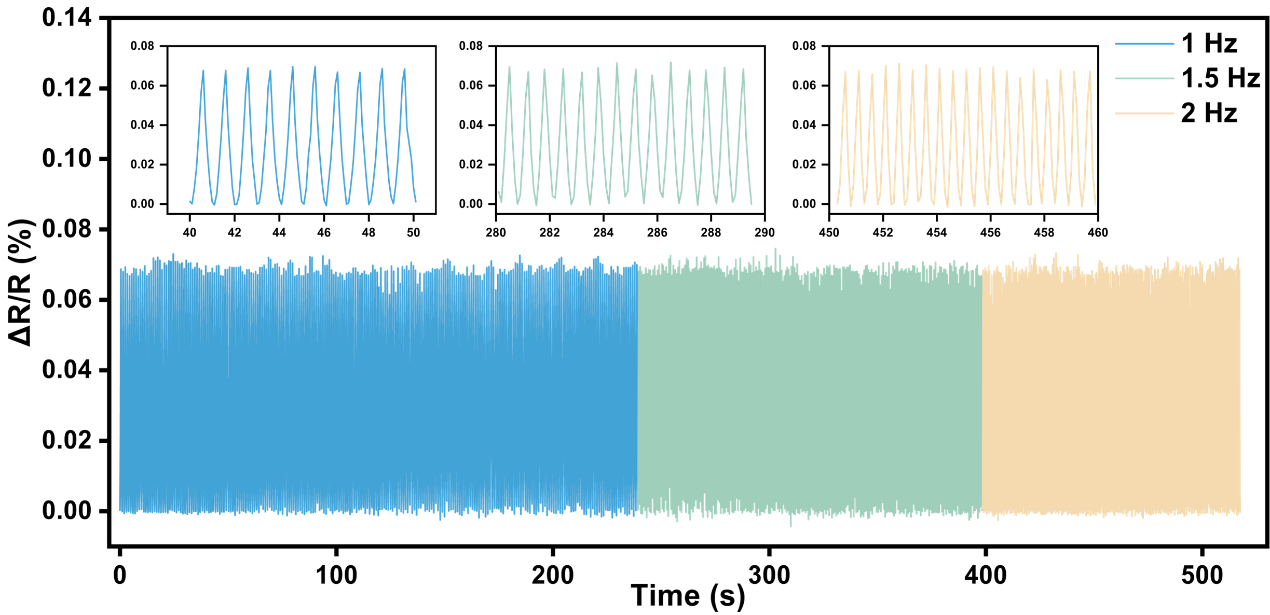


**Fig. S21** Cyclic sensing responses of the PAP hydrogel at 1, 1.5, and 2 Hz under 10% strain


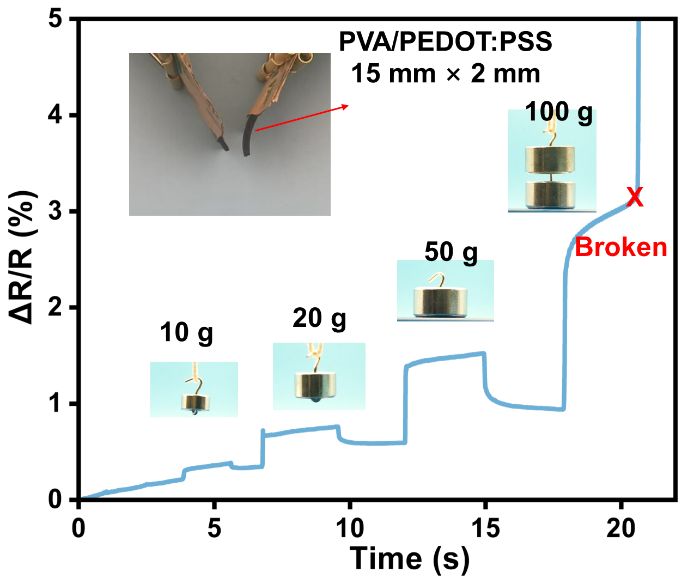


**Fig. S22** Stepwise loading test of the PVA/PEDOT:PSS hydrogel, which fractured at only 100 g, highlighting its poor mechanical robustness


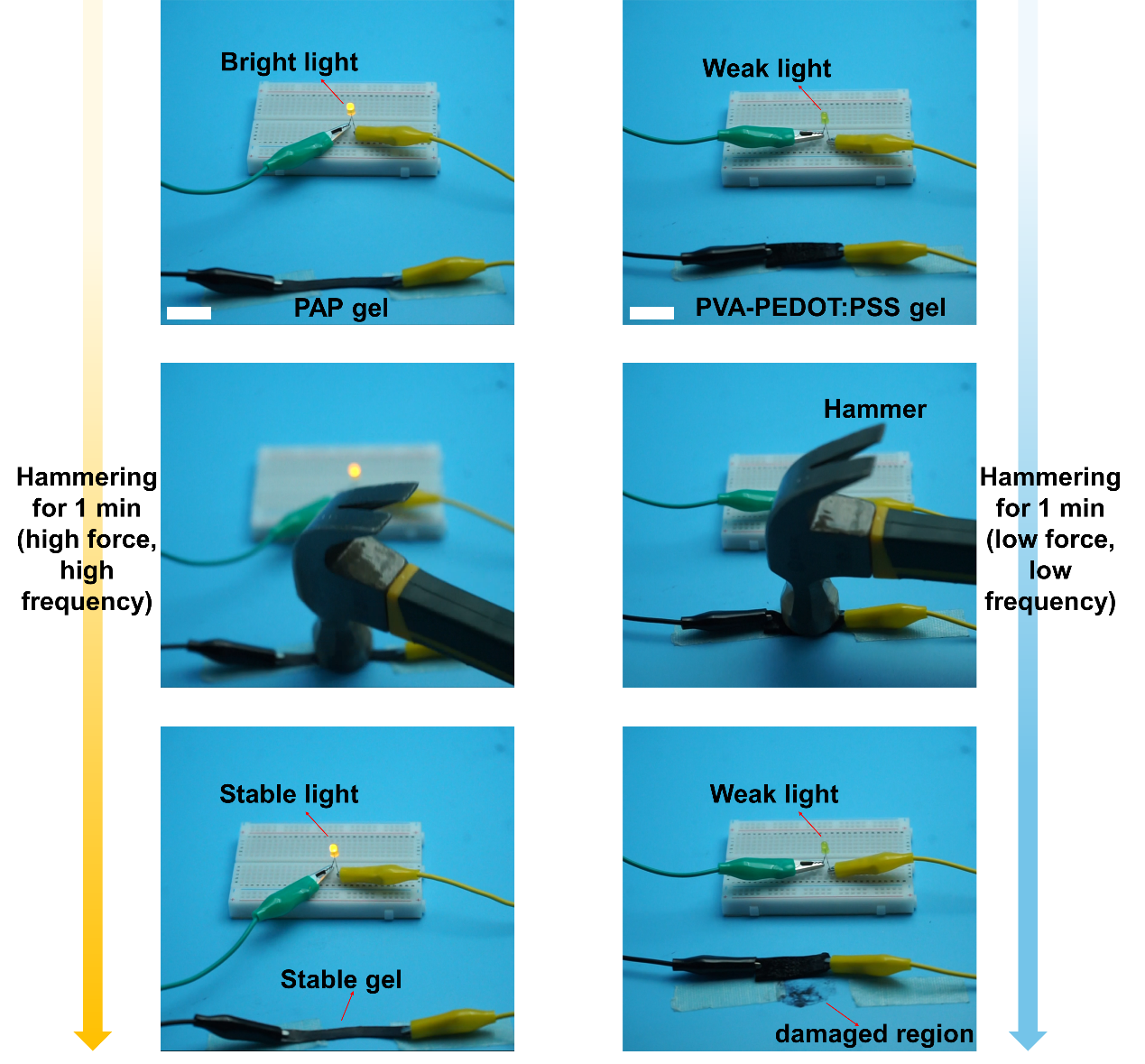


**Fig. S23** Hammering tests of PAP and PVA-PEDOT:PSS hydrogels. PAP hydrogel maintained stable lighting under hammering, while PVA-PEDOT:PSS gel weakened and damaged





**Fig. S24** EMG recordings obtained for 30 minutes per day over three consecutive days using the same hydrogel electrodes


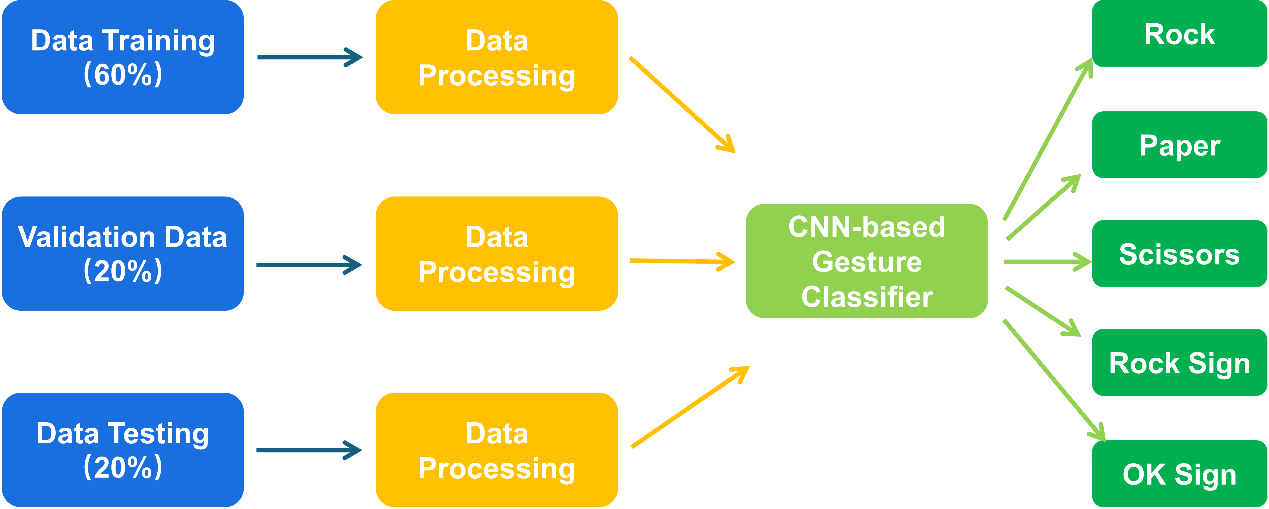


**Fig. S25** Flow chart of the convolutional neural network (CNN)-based machine learning for different finger gesture classification and further recognition

*
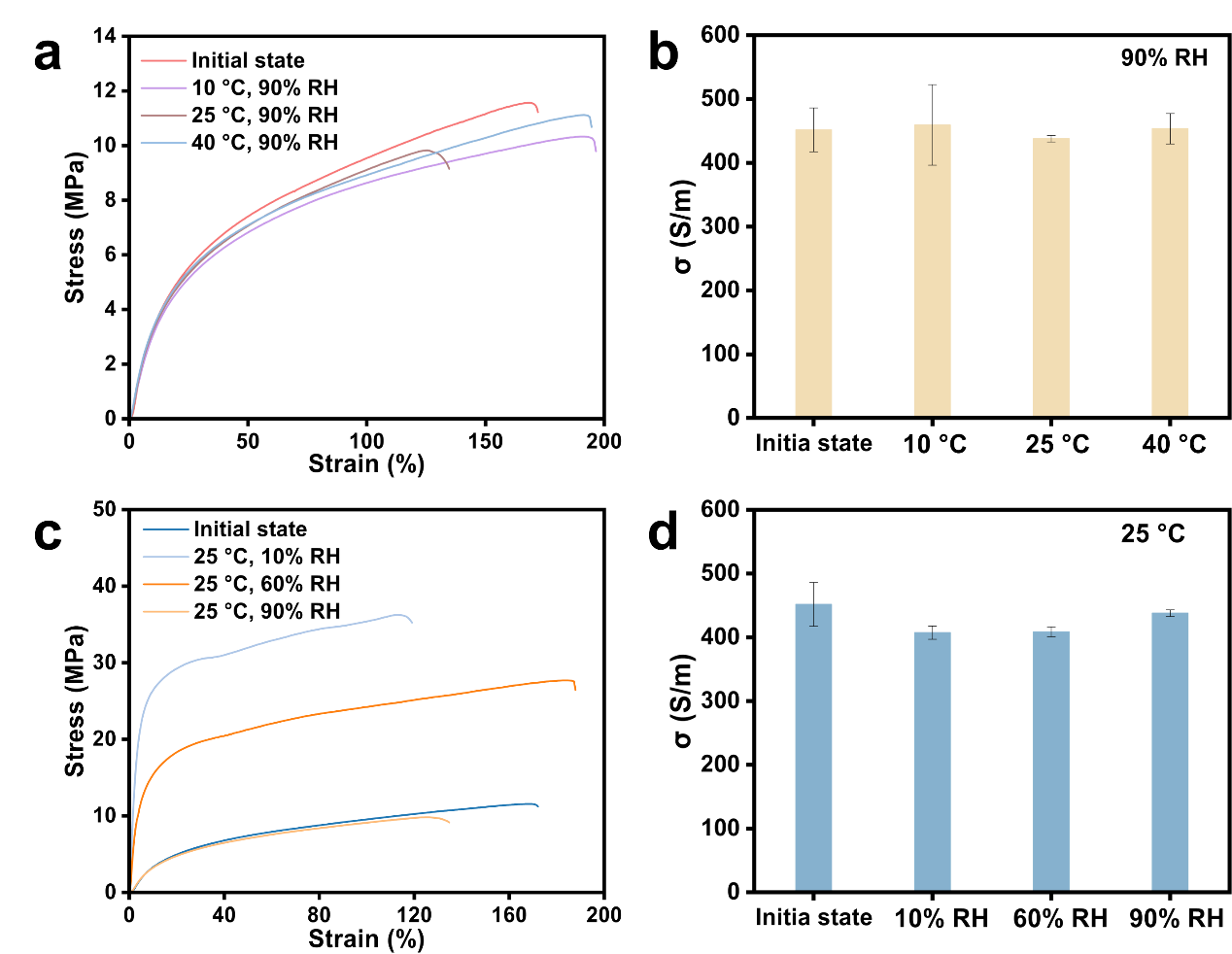
*

**Fig. S26** Mechanical and electrical properties of the PAP hydrogel after 24 h exposure to different temperatures and humidity conditions. **a, c** Tensile stress-strain curves. **b, d** Electrical conductivity. The “initial state” refers to the hydrogel sample taken directly from water and equilibrated at room temperature prior to environmental exposure





**Fig. S27** Time-dependent swelling behavior of PAP hydrogels


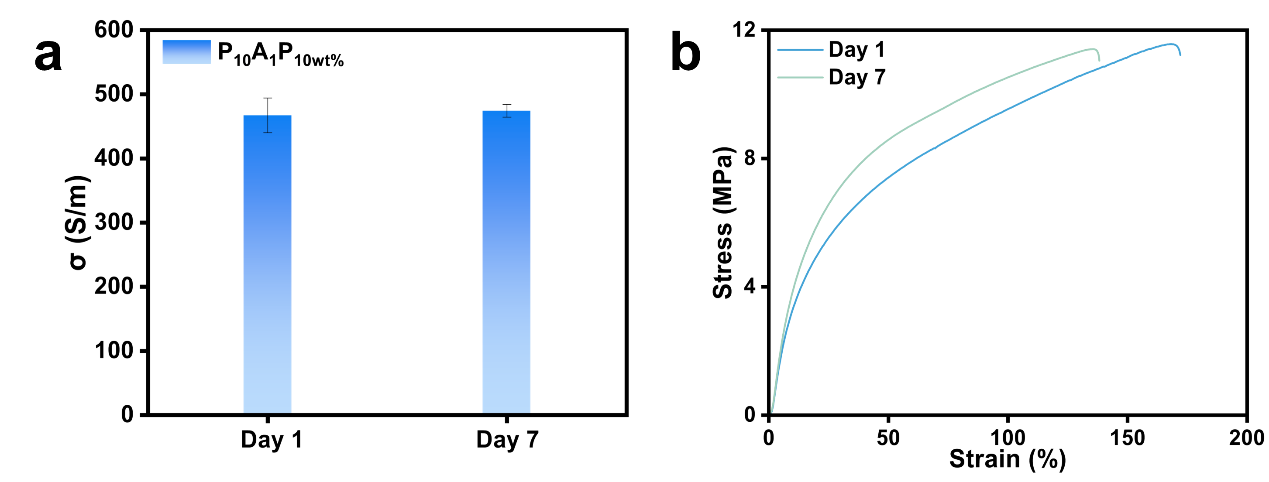


**Fig. S28** Seven-day stability of PAP hydrogels. **a** Electrical conductivity on Day 1 and Day 7. **b** Stress-strain curves on Day 1 and Day 7

*
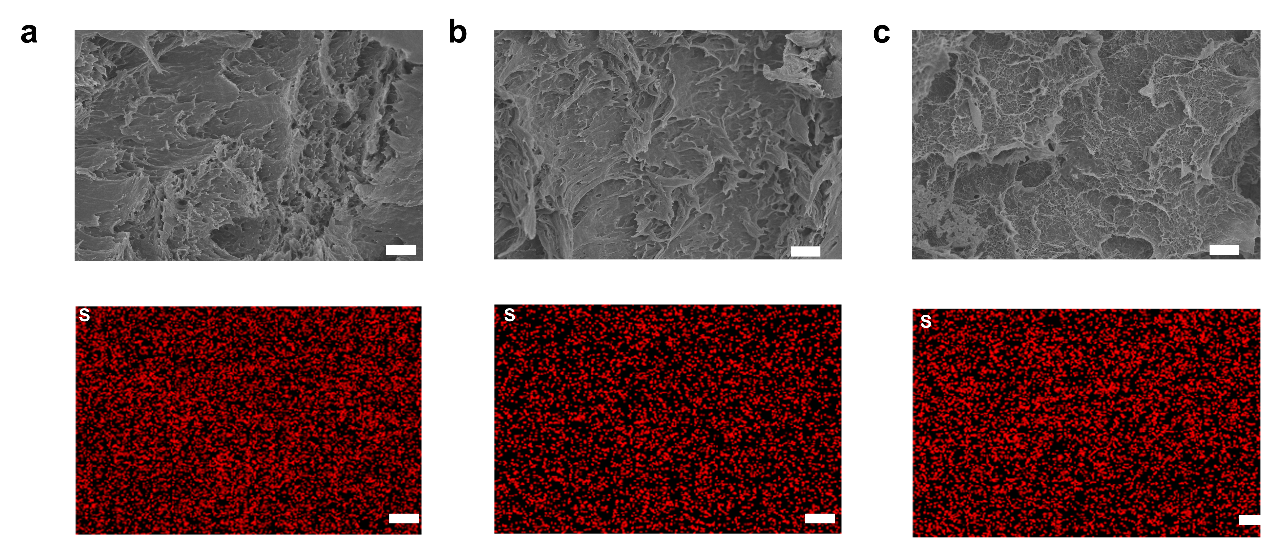
*

**Fig. S29** SEM images and corresponding EDS sulfur (S) elemental mappings of PAP hydrogels in different states: **a** pristine PAP, **b** after cyclic stretching, and **c** after tensile fracture. The S signal remains broadly distributed across the observed regions, indicating that the PEDOT:PSS phase is still present without obvious micron-scale depletion after cyclic loading and fracture

**Table S1** Summary of electrical conductivity, tensile strength, and Young’s modulus of the PVA/ANF/PEDOT:PSS hydrogel in this work compared with previously reported PVA/PEDOT:PSS-based conductive hydrogels

| Refs. | Hydrogels | Conductivity (S m^-1^) | Strength (MPa) | Modulus (kPa) |
| --- | --- | --- | --- | --- |
| This work | PVA/ANF/PEDOT:PSS | 452.75 | 10.72 | 29890 |
| [S1] | PVA/HBPN/PEDOT:PSS | 0.366 | 0.58 | 149.3 |
| [S2] | PVA/SA/PEDOT:PSS | 0.256 | 0.118 | ~ 100 |
| [S3] | PVA/CMC/PEDOT:PSS | 2.04 | 0.391 | 0.09 |
| [S4] | PVA/SBMA/PEDOT:PSS | 0.2 | 1.342 | ~ 81.19 |
| [S5] | PVA/PAA/PEDOT:PSS | 4.43 | 0.035 | 12.79 |
| [S6] | PVA/PEDOT:PSS | 0.5 | 0.038 | ~ 0.5 |
| [S7] | PVA/PEDOT:PSS | 1.38 | 0.55 | 200 |
| [S8] | PVA/PEDOT:PSS/LMNP | 2.8 | 0.26 | 44.93 |
| [S9] | PVA/PEDOT: PSS/MWCNT-COOH | 12.6 | ~ 0.094 | ~ 0.177 |
| [S10] | PVA/Mxene/PEDOT:PSS/RGO/Silk Fibroin | 6.67 | ~ 0.025 | ~ 0.1 |
| [S11] | PPMP-OH Organohydrogel | 0.257 | 1.48 | 211 |
| [S12] | PVA/PEDOT:PSS | 100 | ~ 1.3 | ~ 2.8 |
| [S13] | PVA/PSBMA/PEDOT:PSS | 1.2 | ~ 0.08 | ~ 19.89 |
| [S14] | PVA/PEDOT:PSS/FC γ-PGA/TA | 0.94 | 0.9 | ~ 70.97 |
| [S15] | PVA-SO_4_^2-^/SO_3_^2-^-PEDOT:PSS | 2.92 | 0.833 | 350 |
| [S16] | DFS PVA/PEDOT:PSS | 0.16 | 0.84 | ~ 100 |
| [S17] | PVA/PEDOT:PSS/CTS/  PAA-NHS | > 9 | ~ 2 | 650 |
| [S18] | PVA/PEDOT:PSS | > 9 | ~ 1.8 | ~ 4.2 |
| [S19] | PVA/CNF/PEDOT:PSS | 0.3 | 3.71 | 1.1 |
| [S20] | PVA/PVP/PEDOT:PSS | 90.6 | 5.2 | ~ 55 |
| [S21] | β-CD/citric acid/PVA/PEDOT:PSS | 27 | 0.3 | 223 |
| [S22] | ANF/PPy | 7200 | 27.2  (elongation ~25%) | 512300 |
| [S23] | ANF/PVA/AgNWs | 470 - 1.66 × 104 | 5.5  (elongation ~100%) | 15400 |
| [S24] | MXene/PEDOT:PSS/ANF fibers | ~ 73680 | ~ 160 Mpa  (elongation ~6.9%) | - |
| [S25] | ANFs-PVA-PVP | 0.0105 | 9.7 | 2.17 |
| [S26] | ANFs/PVA/MXene | - | ~ 1.5 MPa | - |
| [S27] | P/CY-ANF | - | 17.9 | 276100 |

**Table S2** Sulfur atomic percentage (%) quantified by EDS from three cross-sectional regions of the PAP hydrogel

| Region | S (%) |
| --- | --- |
| 1 | 1.50 |
| 2 | 1.49 |
| 3 | 1.89 |
| Mean ± SD | 1.63 ± 0.23 |

**Supplementary References**

1. J. Teng, X. Jia, Z. Qiu, H. Yang, H. Li, Amino-ended hyperbranched polyamide-cross-linked conducting polymer hydrogels with enhanced performance for wearable electronics. Nanoscale **17**(15), 9427–9435 (2025). <https://doi.org/10.1039/d4nr05041j>
2. J. Zheng, J. Zhou, Y. Zhao, C. Wang, M. Fan et al., A low-cost hydrogel electrode for multifunctional sensing: strain, temperature, and electrophysiology. Biosensors **15**(3), 177 (2025). <https://doi.org/10.3390/bios15030177>
3. S. Aisyah Nurmaulia Entifar, N. Aqilla Ellenahaya Entifar, A. Fitrian Wibowo, J. Ha Kim, Y. Shara br Sembiring et al., Extremely-low electrical-hysteresis hydrogels for multifunctional wearable sensors and osmotic power generators. Chem. Eng. J. **509**, 160971 (2025). <https://doi.org/10.1016/j.cej.2025.160971>
4. Z. Bi, W. Yuan, Deep-learning-assisted and self-powered hydrogels sensor with high mechanical strength, good biocompatibility and stretchability for information transmission, motion monitoring, health and Huntington’s disease detection. Chem. Eng. J. **507**, 160609 (2025). <https://doi.org/10.1016/j.cej.2025.160609>
5. X. Zhang, D. Li, G. Liu, 3D printed ultrasoft and adhesive PEDOT: PSS-based hydrogel for bioelectronics. ACS Appl. Polym. Mater. **7**(3), 1531–1539 (2025). <https://doi.org/10.1021/acsapm.4c03275>
6. A. Khadka, S. Pradhan, E. Samuel, B. Joshi, H. Gao et al., Rapidly self-healing, highly conductive, stretchable, body-attachable hydrogel sensor for soft electronics. Compos. Commun. **52**, 102158 (2024). <https://doi.org/10.1016/j.coco.2024.102158>
7. G. Li, R. Wan, S. Liu, L. Wang, M. Yu et al., High-performance multipedal shape strain sensors for human motion and electrophysiological signal monitoring. Macromol. Chem. Phys. **225**(22), 2400224 (2024). <https://doi.org/10.1002/macp.202400224>
8. K. Zhao, Y. Zhao, J. Xu, R. Qian, Z. Yu et al., Stretchable, adhesive and self-healing conductive hydrogels based on PEDOT: PSS-stabilized liquid metals for human motion detection. Chem. Eng. J. **494**, 152971 (2024). <https://doi.org/10.1016/j.cej.2024.152971>
9. L. Zhao, C. Fang, B. Qin, X. Yang, P. Poechmueller, Conductive dual-network hydrogel-based multifunctional triboelectric nanogenerator for temperature and pressure distribution sensing. Nano Energy **127**, 109772 (2024). <https://doi.org/10.1016/j.nanoen.2024.109772>
10. Z. Zheng, W. Xu, Y. Wang, W. Xiong, C. Xiong et al., High-conductivity and long-term stability strain sensor based on silk fibroin and polyvinyl alcohol hydrogels. Mater. Today Commun. **38**, 108465 (2024). <https://doi.org/10.1016/j.mtcomm.2024.108465>
11. D. Bi, N. Qu, W. Sheng, T. Lin, S. Huang et al., Tough and strain-sensitive organohydrogels based on MXene and PEDOT/PSS and their effects on mechanical properties and strain-sensing performance. ACS Appl. Mater. Interfaces **16**(9), 11914–11929 (2024). <https://doi.org/10.1021/acsami.3c18631>
12. Y.-W. Kim, J.-M. Park, C.S. Park, H. Na, Y.-W. Kang et al., Anisotropically conductive hydrogels with directionally aligned PEDOT: PSS in a PVA matrix. ACS Appl. Mater. Interfaces **16**(3), 4013–4023 (2024). <https://doi.org/10.1021/acsami.3c16094>
13. J. Yu, R. Wan, F. Tian, J. Cao, W. Wang et al., 3D printing of robust high-performance conducting polymer hydrogel-based electrical bioadhesive interface for soft bioelectronics. Small **20**(19), e2308778 (2024). <https://doi.org/10.1002/smll.202308778>
14. C. Gao, D. Zheng, B. Long, Z. Chen, J. Zhu et al., Anti-swelling and adhesive γ-PGA/PVA/PEDOT: PSS/TA composite conductive hydrogels for underwater wearable sensors. Eur. Polym. J. **201**, 112590 (2023). <https://doi.org/10.1016/j.eurpolymj.2023.112590>
15. C. Tian, C. Bai, T. Wang, Z. Yan, Z. Zhang et al., Thermogalvanic hydrogel electrolyte for harvesting biothermal energy enabled by a novel redox couple of SO_4/3_ ^2-^ ions. Nano Energy **106**, 108077 (2023). <https://doi.org/10.1016/j.nanoen.2022.108077>
16. Z. Zhang, G. Chen, Y. Xue, Q. Duan, X. Liang et al., Fatigue-resistant conducting polymer hydrogels as strain sensor for underwater robotics. Adv. Funct. Mater. **33**(42), 2305705 (2023). <https://doi.org/10.1002/adfm.202305705>
17. F. Wang, Y. Xue, X. Chen, P. Zhang, L. Shan et al., 3D printed implantable hydrogel bioelectronics for electrophysiological monitoring and electrical modulation. Adv. Funct. Mater. **34**(21), 2314471 (2024). <https://doi.org/10.1002/adfm.202314471>
18. Y. Xue, X. Chen, F. Wang, J. Lin, J. Liu, Mechanically-compliant bioelectronic interfaces through fatigue-resistant conducting polymer hydrogel coating. Adv. Mater. **35**(40), e2304095 (2023). <https://doi.org/10.1002/adma.202304095>
19. N. Li, Q. Yu, S. Duan, Y. Du, X. Shi et al., Anti-swelling, high-strength, anisotropic conductive hydrogel with excellent biocompatibility for implantable electronic tendon. Adv. Funct. Mater. **34**(12), 2309500 (2024). <https://doi.org/10.1002/adfm.202309500>
20. Q. Han, X. Gao, C. Zhang, Y. Tian, S. Liang et al., Acid-induced *in situ* phase separation and percolation for constructing bi-continuous phase hydrogel electrodes with motion-insensitive property. Adv. Mater. **37**(6), e2415445 (2025). <https://doi.org/10.1002/adma.202415445>
21. S. Guo, S. Zhang, H. Li, S. Liu, J.J. Koh et al., Precisely manipulating polymer chain interactions for multifunctional hydrogels. Matter **8**(4), 101785 (2025). <https://doi.org/10.1016/j.matt.2024.06.024>
22. H. He, Y. Chen, A. Pu, L. Wang, W. Li et al., Strong and high-conductivity hydrogels with all-polymer nanofibrous networks for applications as high-capacitance flexible electrodes. npj Flex. Electron. **8**, 56 (2024). <https://doi.org/10.1038/s41528-024-00346-8>
23. Q. Zhou, J. Lyu, G. Wang, M. Robertson, Z. Qiang et al., Mechanically strong and multifunctional hybrid hydrogels with ultrahigh electrical conductivity. Adv. Funct. Mater. **31**(40), 2104536 (2021). <https://doi.org/10.1002/adfm.202104536>
24. S. Wang, X. Guo, S. Liao, J. Chen, Q. Wei, Aramid nanofiber-reinforced MXene/PEDOT: PSS hybrid fibers for high-performance fiber-shaped supercapacitors. Electrochim. Acta **466**, 143062 (2023). <https://doi.org/10.1016/j.electacta.2023.143062>
25. D. Ji, Z. Zhang, J. Sun, W. Cao, Z. Wang et al., Strong, Tough, and Biocompatible Poly(vinyl alcohol)-Poly(vinylpyrrolidone) Multiscale Network Hydrogels Reinforced by Aramid Nanofibers. ACS Appl. Mater. Interfaces **16**(19), 25304–25316 (2024). <https://doi.org/10.1021/acsami.4c02354>
26. C. Wang, Z. Zhu, L. Han, L. Xu, M. Wang et al., Poly(vinyl alcohol) hydrogels enhanced with Ti_3_C_2_T*_x_* MXene nanosheets and aramid nanofibers for electromagnetic shielding and motion detection. ACS Appl. Nano Mater. **7**(21), 24925–24937 (2024). <https://doi.org/10.1021/acsanm.4c04840>
27. H.J. Kim, H. Kim, Y.H. Choi, E.S. Lee, Y.H. Kim et al., Rapid fabrication of tendon-inspired ultrastrong, water-rich hydrogel fibers: synergistic engineering of cyano-p-aramid nanofibers and poly(vinyl alcohol). ACS Nano **19**(8), 8316–8327 (2025). <https://doi.org/10.1021/acsnano.4c18686>
